# A Comparison of Markov and Mechanistic Models for Soil-Transmitted Helminth Prevalence Projections in the Context of Survey Design

**DOI:** 10.1093/cid/ciae022

**Published:** 2024-04-25

**Authors:** Max T Eyre, Caroline A Bulstra, Olatunji Johnson, Sake J de Vlas, Peter J Diggle, Claudio Fronterrè, Luc E Coffeng

**Affiliations:** Centre for Health Informatics, Computing and Statistics, Lancaster Medical School, Lancaster University, Lancaster, United Kingdom; Department of Disease Control, Faculty of Infectious and Tropical Diseases, London School of Hygiene and Tropical Medicine, London, United Kingdom; Department of Public Health, Erasmus MC, University Medical Center Rotterdam, Rotterdam, The Netherlands; Heidelberg Institute of Global Health (HIGH), Heidelberg University Medical Center, Heidelberg, Germany; Department of Mathematics, University of Manchester, Manchester, United Kingdom; Department of Public Health, Erasmus MC, University Medical Center Rotterdam, Rotterdam, The Netherlands; Centre for Health Informatics, Computing and Statistics, Lancaster Medical School, Lancaster University, Lancaster, United Kingdom; Centre for Health Informatics, Computing and Statistics, Lancaster Medical School, Lancaster University, Lancaster, United Kingdom; Department of Public Health, Erasmus MC, University Medical Center Rotterdam, Rotterdam, The Netherlands

**Keywords:** geostatistics, Markov model, prevalence surveys, soilt-transmitted helminths, transmission model

## Abstract

Globally, there are over 1 billion people infected with soil-transmitted helminths (STHs), mostly living in marginalized settings with inadequate sanitation in sub-Saharan Africa and Southeast Asia. The World Health Organization recommends an integrated approach to STH morbidity control through improved access to sanitation and hygiene education and the delivery of preventive chemotherapy (PC) to school-age children delivered through schools. Progress of STH control programs is currently estimated using a baseline (pre-PC) school-based prevalence survey and then monitored using periodical school-based prevalence surveys, known as Impact Assessment Surveys (IAS). We investigated whether integrating geostatistical methods with a Markov model or a mechanistic transmission model for projecting prevalence forward in time from baseline can improve IAS design strategies. To do this, we applied these 2 methods to prevalence data collected in Kenya, before evaluating and comparing their performance in accurately informing optimal survey design for a range of IAS sampling designs. We found that, although both approaches performed well, the mechanistic method more accurately projected prevalence over time and provided more accurate information for guiding survey design. Both methods performed less well in areas with persistent STH hotspots where prevalence did not decrease despite multiple rounds of PC. Our findings show that these methods can be useful tools for more efficient and accurate targeting of PC. The general framework built in this paper can also be used for projecting prevalence and informing survey design for other neglected tropical diseases.

## Teaser

Globally, over 1 billion people have STH infections. Monitoring STH control programs involves baseline and periodic Impact Assessment Surveys (IAS). This paper shows that integrating geostatistical methods with transmission models enhances IAS design, aiding more effective preventive chemotherapy targeting.

Soil-transmitted helminths (STHs) are parasitic intestinal nematodes that are transmitted between humans through contaminated soil and are composed of *Ascaris lumbricoides*, *Trichuris trichiura*, and hookworm spp. (*Ancylostoma duodenale* and *Necator americanus*). These 4 species are considered together because of their similar transmission dynamics, diagnosis, control, and prevention measures. It is common for a single individual, particularly children in impoverished settings, to be chronically infected with more than 1 species at the same time [1, 2]. Globally, there are over 1 billion people infected with STHs, one of the most common neglected tropical diseases (NTDs) worldwide. The majority of cases are found in marginalized settings with inadequate sanitation in sub-Saharan Africa and Southeast Asia and present a major public health burden globally [3].

The World Health Organization (WHO) recommends an integrated approach to STH morbidity control, through improved access to sanitation and hygiene education and the school-based delivery of preventive chemotherapy (PC) with albendazole or mebendazole to school-age children (SAC). It has set a target of reducing the prevalence of moderate- and heavy-intensity infections below 2% in SAC and pre–school-age children by 2030 [4]. Typically, STH prevalence is initially estimated using a baseline (pre-PC) school-based prevalence survey of SAC, conducted at a number of selected primary schools in endemic areas. Soil-transmitted helminth control progress is then monitored using periodical school-based prevalence surveys, known as Impact Assessment Surveys (IAS). The IAS are typically carried out after 5 years of PC and are used to estimate current STH prevalence at the implementation unit (IU) level to inform decisions on the requirements for PC delivery, with the aim of reaching elimination as a public health problem. When the prevalence of STHs (any intensity) in the target population falls below 2%, the WHO recommends suspending PC [5].

In the context of limited financial resources in STH-endemic countries and the high cost associated with conducting prevalence surveys, there is a need for careful design of surveys to accurately and efficiently measure prevalence burden and capture geographical variation in prevalence. Our previous work applying model-based geostatistical methods to this problem has demonstrated that they can significantly increase the precision of prevalence surveys relative to traditional survey design, thus reducing field-sampling effort while maintaining or improving precision [6, 7]. The aim of this study was to investigate whether integrating geostatistical methods with Markov or mechanistic models can accurately project prevalence forward in time and help improve IAS design. While this study focuses on STH impact surveys, the methodology and principles are applicable to post-baseline survey design for other NTDs.

## METHODS

### Data

#### Soil-Transmitted Helminth Prevalence and Preventive Chemotherapy Coverage Data

**Figure 1.**
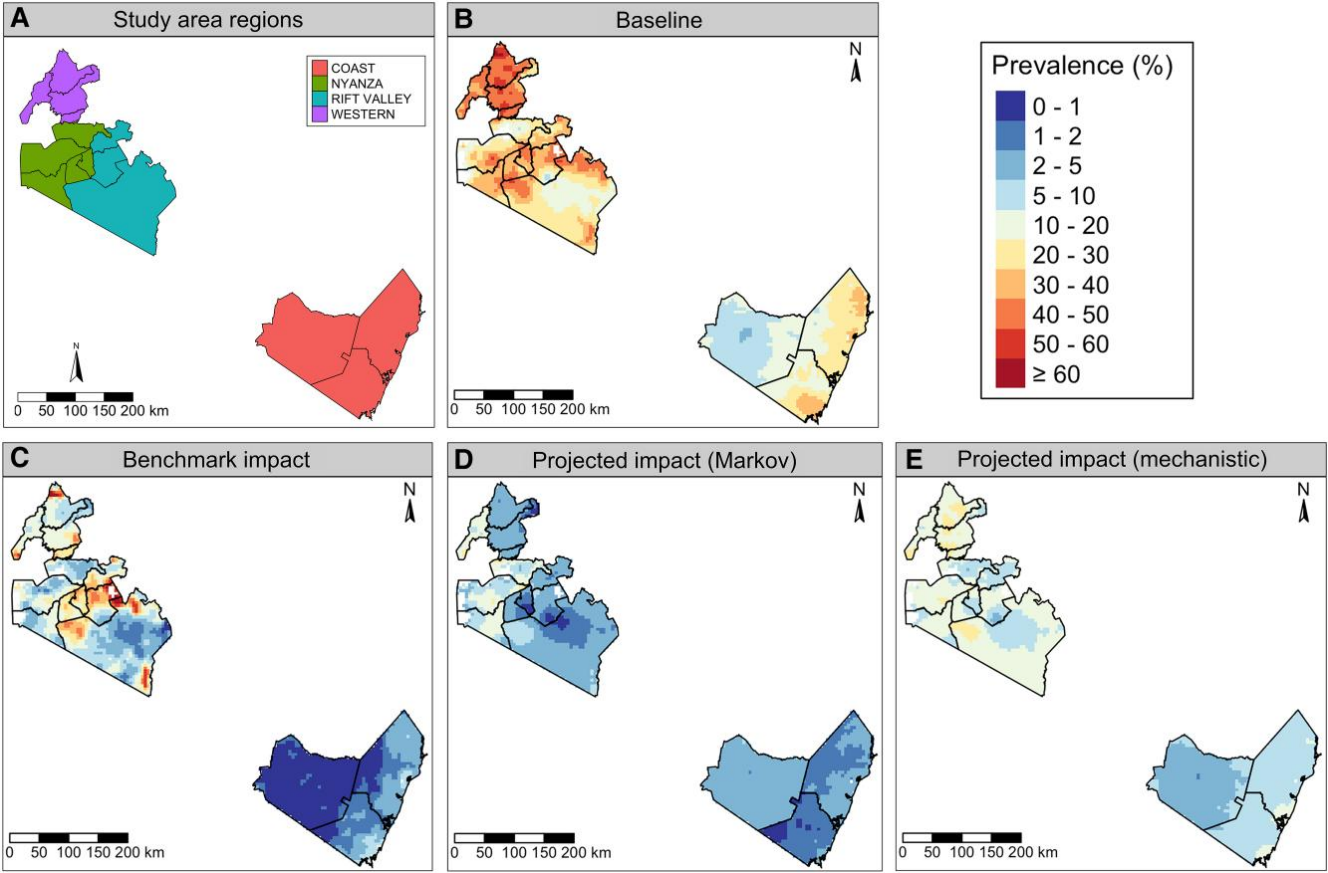
*A*, Study area in southwest Kenya, consisting of 16 implementation units (boundaries indicated by black lines) within 4 regions: Coast, Nyanza, Rift Valley, and Western. *B* and *C*, STH prevalence in SAC as predicted by the geostatistical model fit to baseline and impact prevalence survey data (the prediction surface at impact is used as our benchmark). *D* and *E*, STH prevalence projected at impact using the Markov and mechanistic approaches. Abbreviations: SAC, school-age children; STH, soil-transmitted helminth.

Soil-transmitted helminth prevalence and PC coverage data were collected in 16 IUs (districts, administrative level 2) in Kenya (see Figure 1*A* ) to monitor the reduction in STH infection in response to annual PC for SAC during a national school-based deworming program (NSBDP) between 2012 and 2017 [8]. Estimated PC coverage in each round was based on pre-PC surveys carried out approximately 1 year after each previous PC round and 2–5 weeks before the start of the next PC round and were recorded for each IU (see Supplementary Figure 1). These data are publicly available via the Global Atlas of Helminth Infections (https://www.thiswormyworld.org/ ) and the ESPEN portal (https://espen.afro.who.int/ ).

The NSBDP study design (described in more detail previously [8]) consisted of repeat cross-sectional surveys in a representative, population-stratified random sample of 172 schools across the 16 IUs at 3 time points over a 5-year period: baseline (pre-PC, 2012), midterm (after 2 rounds of PC, 2015), and impact (after 4 rounds of PC, 2017). During each survey, stool samples were collected from a randomly selected sample of approximately 100 schoolchildren at each school and tested for the presence of each STH species using the Kato-Katz thick smear technique.

#### Environmental and Demographic Data

Environmental data for the study area were available from moderate resolution imaging spectroradiometer [9, 10] and consisted of rasters at 5-km resolution for the following variables: elevation, Enhanced Vegetation Index (EVI), mean daytime land surface temperature (LST), mean nighttime LST, Normalized Difference Vegetation Index, soil acidity, and soil moisture. Population density data at 1-km resolution was used from WorldPop [11].

### Overview of Study Analysis Steps

This study consisted of 3 main steps (see Supplementary Figure 2 for a diagram), as discussed in the following sections.

#### Step 1. Geostatistical Modeling of Survey Data

First, we fitted independent binomial geostatistical models to baseline prevalence survey data for each of the 3 STH species (*A. lumbricoides*, *T. trichiura*, and hookworm spp.) and used them to predict baseline prevalence at the pixel level (5-km resolution). To improve model precision, we explored the use of a set of spatially varying environmental covariates that are known to be potential drivers of STH transmission and included EVI, mean daytime LST, mean nighttime LST, and soil acidity in the model because they had an approximately linear relationship with prevalence for each of the 3 species on the log-odds scale (Supplementary Figures 3–5). A detailed explanation of the geostatistical modelling process is provided in Supplementary Section 1 and model parameter estimates are shown in Supplementary Table 1, respectively. These true baseline species-specific prevalence surfaces were then used as the input for the 2 prevalence projecting methods in Step 2.

To create a post-PC benchmark to evaluate the performance of the 2 projecting methods we followed the same methodology to fit binomial geostatistical models to actual observed post-PC prevalence impact survey data and predict species-specific prevalence surfaces, which were then aggregated across species, assuming the risk of an individual contracting each was independent. The IU-level prevalence was then classified into 5 endemicity classes (0–2%, 2–10%, 10–20%, 20–50%, and 50–100%), taking into account population density. Model covariate relationship plots and model parameter estimates are included in Supplementary Figures 6–8 and Supplementary Table 2. These predicted outputs are what we try to reproduce using the 2 methods in Step 2.

#### Step 2. Projecting Prevalence Surface to Impact

In Step 2, we projected the baseline prevalence surfaces forward to the time of the proposed impact survey using 2 different approaches: a multistate Markov model [12] and a mechanistic transmission model, WORMSIM [13]. The mechanistic approach only used the baseline prevalence surface and PC coverage data to achieve this, whereas the Markov model also required midterm prevalence survey data (collected after 2 rounds of PC in 2015).

##### Method 1: Multistate Markov Model

This method followed a 2-stage procedure, described in more detail previously [12]. We first fitted a multistate Markov model (for each species independently) to baseline and midterm school prevalence data that were grouped into 5 prevalence categories (0–2%, 2–10%, 10–20%, 20–50%, and 50–100%) to estimate regression coefficients for the effect of baseline-midterm PC history on the probability of transition in prevalence category at the school level. We then used these coefficients to predict the transition in endemicity class (also categorized as 0–2%, 2–10%, 10–20%, 20–50%, and 50–100%) for each IU between baseline and impact using baseline-impact PC history. Finally, to generate a projected impact prevalence surface we performed a local scaling of the predicted baseline prevalence surface, such that the population-weighted mean prevalence of the surface is equal to the endemicity class estimated by the Markov model (on the log-odds scale) and then aggregate across species, assuming independence.

##### Method 2: Mechanistic Transmission Modeling With WORMSIM

WORMSIM is an established individual-based stochastic model for transmission and control of helminth infections in humans [13], which simulates the life histories of individual humans and individual worms within a closed human population. A formal description of WORMSIM with extensive technical details and mathematical formulae has been published previously [13, 14]; the main aspects are described in Supplementary Section 2. We used WORMSIM to simulate the impact of PC on STH prevalence among SAC in each of the 16 IUs within our study area, based on IU-level baseline prevalence data and PC coverage levels (shown for each IU in Supplementary Figure 2). The projected impact prevalence surface was then generated using the same local scaling methodology previously described.

#### Step 3. Simulation Study to Compare Survey Design Performance

Finally, we conducted a Monte Carlo simulation study to compare the performance of simulated survey designs based on 3 prevalence surfaces: the 2 projected impact prevalence surfaces (Step 2) and the benchmark impact surface (Step 1). We evaluated the performance of 24 survey design scenarios in terms of cost and accuracy in determining IU endemicity class (compared with the “true” benchmark prevalence surface at impact from Step 1). The candidate survey designs were created by varying the following: (1) the number of schools to sample (we sampled 20%, 30%, 40%, 60%, 80%, and 100% of the total 172 schools used in the original impact survey) and (2) the number of children per school (we considered values of 30, 50, 70, and 100).

We then followed the following simulation process. First, for a given design scenario and prevalence surface, the chosen number of schools were randomly sampled from 9511 geo-referenced schools within the study area. Second, prevalence survey data for each STH species were simulated at each school as a realization of a binomial random variable with probability equal to the predicted prevalence at the school's location for the given surface and number of trials equal to the number of children per school. Third, 3 independent binomial geostatistical models were fitted to the simulated school data for each STH species with the same 5 covariates used in Step 1. Fourth, predicted prevalence surfaces were predicted from the fitted geostatistical models for each of the 3 species and then combined to create a joint population-weighted “any STH” prevalence surface. Fifth, for each IU, we drew samples from the predictive distribution of the IU-wide population-weighted “any STH” prevalence and calculated the predictive probability of belonging to each of the 5 endemicity classes. The endemicity class with the highest probability was then assigned to the IU. This was repeated 1000 times for each of the 24 survey designs for each of the 3 prevalence surfaces.

We then evaluated the performance of each survey design by calculating the proportion of correctly classified IUs. The benchmark for performance for each of the 3 surfaces was the IU-level endemicity class classified from each projected surface (from which the synthetic school prevalence data were simulated) using a probabilistic classification algorithm for IUs developed previously [6].

## RESULTS

### Multistate Markov Predictions

Prevalence predictions from the multistate Markov model for each IU and STH species are plotted against the modeled prevalence at impact (our benchmark) in Supplementary Figure 9. The Markov model generally performed better for lower prevalence IUs, predicting hookworm spp. and *T. trichiura* prevalence moderately well. For *A. lumbricoides*, however, it significantly underestimated prevalence for the majority of IUs, predicting values in the 0–2% prevalence category for IUs with prevalence rates in excess of 5%.

### WORMSIM Predictions

Four different models were developed for each of the species (see Supplementary Figures 10–15) and the final models for each species were chosen based on simulated baseline prevalence and expected effectivity of school-based PC in SAC. The selected model for the different species is presented as model 1. For *A. lumbricoides* for IUs with higher baseline prevalence levels, the best-fitting model generally predicted a lower impact prevalence than has been observed in the data, suggesting that PC was less effective for decreasing prevalence levels. For *T. trichiura*, the predictions of the best-fitting model were accurate, closely fitting values observed in the data at impact in 12 out of 16 IUs. For hookworm spp., the best-fitting model was able to predict impact prevalence of hookworm spp. relatively accurately. However, the benchmark impact prevalence levels were always in the lower range of the predicted fluctuations in prevalence over time, the opposite of what would be expected (that impact prevalence would be on the higher ends of the ranges) due to sampling taking place directly before the next round of PC took place.

### Projected Prevalence Surfaces at Impact

Projected prevalence surfaces for the Markov and mechanistic models were generated by scaling the baseline prevalence surface by each model's IU-level prevalence predictions. The projected surfaces of *A. lumbricoides* prevalence at impact for both the Markov and mechanistic models (Supplementary Figure 16) failed to capture the hotspots above 10% prevalence in Nyanza and Rift Valley regions (see Figure 1*A* for study area map), although the mechanistic projected surface performed better in this area, predicting prevalence in the range of 5–10%. In contrast, the Markov projected surface consistently predicted prevalence in the range of 0–1% for this area. This is likely to be because prevalence in these areas did not decline significantly between baseline and impact, suggesting a limited impact of PC. The projected prevalence surfaces for both models capture the low prevalence areas (0–5%) well, but the mechanistic projected surface slightly overestimated prevalence in the very low (0–1%) prevalence Coast region.

For *T. trichiura*, the Markov projected surface generally underestimated prevalence and missed the hotspots in the Nyanza and Rift Valley regions (Supplementary Figure 17). In contrast, the mechanistic projected surface captured hotspots but tended to overestimate prevalence in the lowest prevalence areas of the Rift Valley. Projected surfaces for both models overestimated prevalence in the Coast region where the predictions from the geostatistical model at impact indicated that prevalence had fallen to very low levels (0–1%).

For hookworm spp., the projected surfaces for both models performed well, although the mechanistic projections overestimated prevalence in the Coast, Nyanza, and Western regions (Supplementary Figure 18). The improved performance for the projected surfaces of both models for hookworm spp. relative to the other 2 species appeared to be driven by the consistent reduction in prevalence between baseline and impact across all IUs, with an absence of any persistent hotspots.

Both the Markov and mechanistic projected surfaces captured the reduction in STH prevalence in the 3 IUs in the Coast region, although they both slightly overestimated prevalence in the western IU in the region. The high prevalence area (20–60%) in the middle of the Nyanza and Rift Valley regions was missed by both models, although the mechanistic projected surface predicted slightly higher prevalence (10–30%) than the Markov surface (0–20%). The Markov projected surface underestimated the prevalence in the Western region (2–5%), whereas the mechanistic projections more accurately predicted the true prevalence in this area.

**Figure 2.**
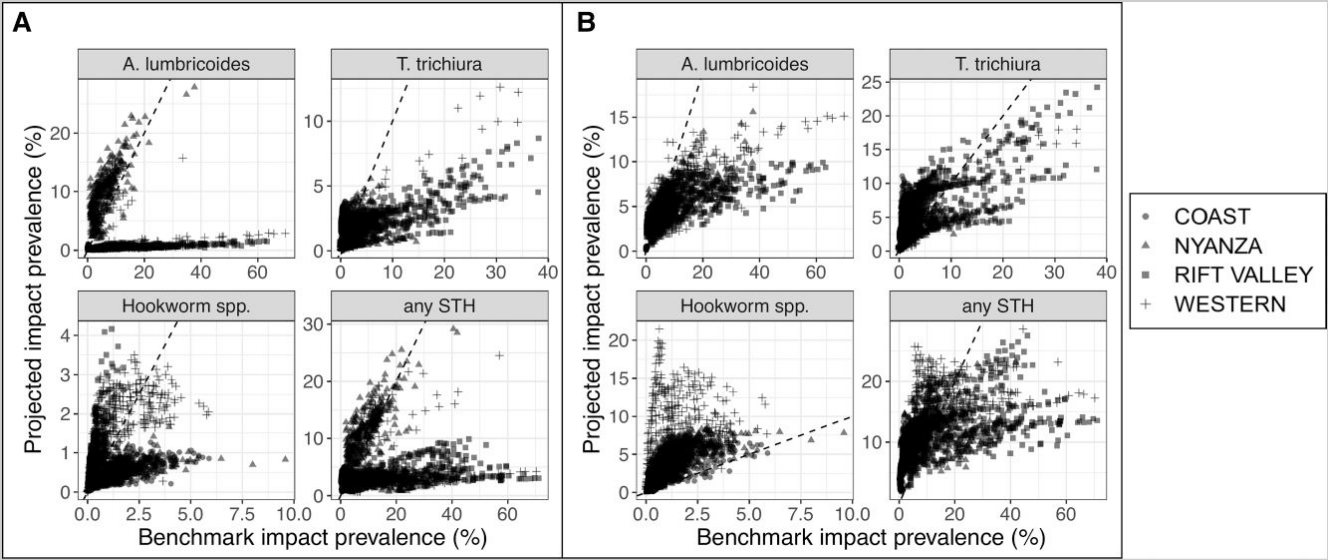
Prevalence predictions at impact projected by the Markov (*A*) and mechanistic (*B*) models compared with the benchmark values predicted by the geostatistical model fit to impact survey data, shown for each STH species with geographic region depicted by point shape (each point represents a pixel within the study area). Abbreviation: STH, soil-transmitted helminth.

The Markov projected surface predicted *A. lumbricoides* and *T. trichiura* prevalence in the Coast and Nyanza regions accurately (Figure 2*A* ), but consistently predicted lower prevalence values in the Rift Valley region and some areas of the Western region. For hookworm spp. the regional differences were less profound, with some areas of the Coast and Nyanza regions having lower predicted values than the benchmark values. The mechanistic projected surface predicted *A. lumbricoides* and *T. trichiura* prevalence at impact more accurately at lower prevalence levels (Figure 2*B* ), but generally tended to underestimate prevalence at higher modeled prevalence levels. However, in the Coast region, the mechanistic projections overestimated *T. trichuria* prevalence. For hookworm spp., the mechanistic projections consistently overestimated prevalence, with this being most pronounced in the Western region.

### Survey Simulation Results

**Figure 3.**
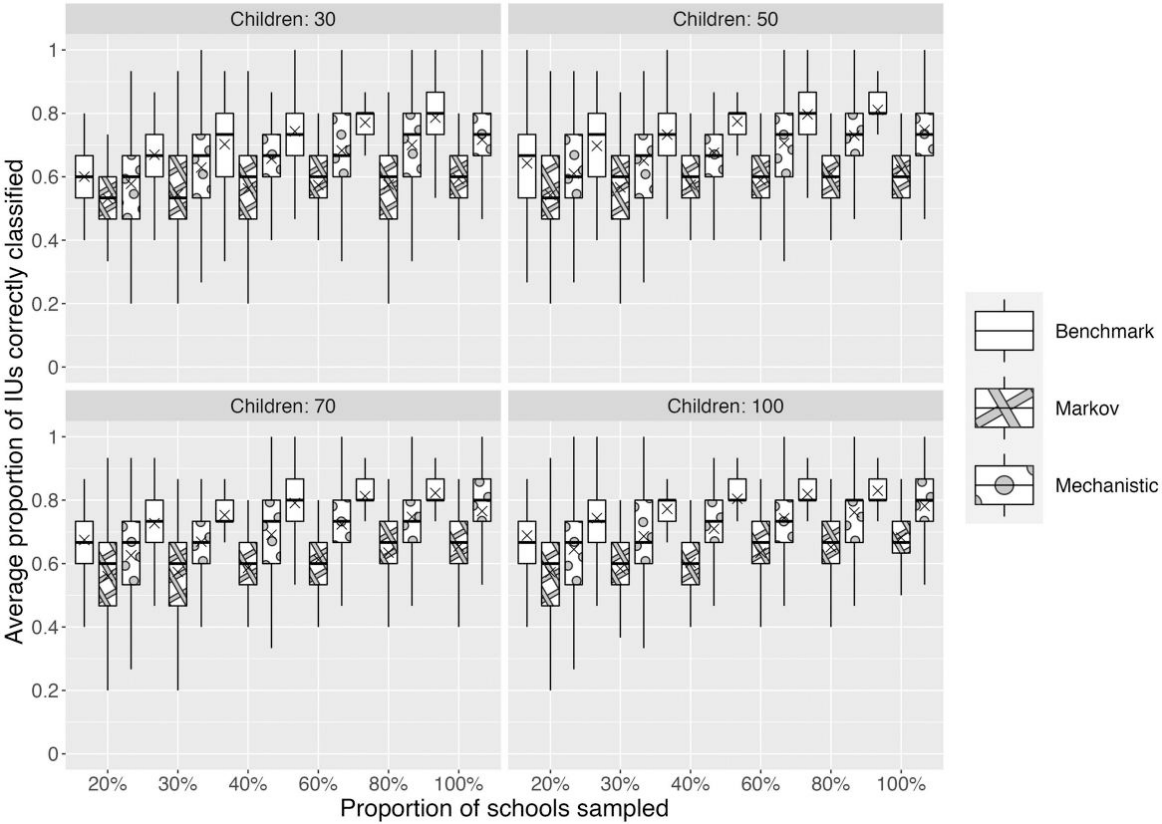
Comparison of the performance (percentage of IUs with the endemicity level correctly classified) of a range of survey designs (for varying values of the number of children per school and proportion of schools sampled) for the benchmark geostatistical model predictions from the impact survey data (ie, the best-available information), and the Markov and mechanistic projected surfaces. Abbreviation: IU, implementation unit.

There was significant variation in the outcome across simulations for each survey design. Figure 3 shows how the proportion of IUs that were correctly classified in terms of endemicity class increased with a higher number of children sampled in each school and a higher proportion of schools sampled. Compared with the performance of simulated surveys based on the best-available information (ie, the predicted surface from the geostatistical model fitted to the impact data), surveys based on the mechanistic and Markov model projected surfaces generally performed worse. The performance of surveys based on the Markov projected surface was consistently between 10 and 20 percentage points lower, while the performance of survey designs based on the mechanistic model were similar to or, at most, 7 percentage points lower than the benchmark.

## DISCUSSION

In this study we compared 2 approaches for projecting STH prevalence at impact that integrate model-based geostatistical predictions of baseline prevalence and model-based forward predictions of prevalence using (1) WORMSIM, a mechanistic transmission model, and (2) multistate Markov models for school-level prevalence categories. We then evaluated their performance using STH prevalence data from Kenya. This is the first study to have directly compared a mechanistic and a more empirical Markov model for projecting prevalence during PC in the context of guiding decisions on IAS design.

We found that, while the Markov and mechanistic approaches both generally performed well for projecting STH prevalence at impact, prediction accuracy was lower in areas with persistent high prevalence hotspots that were not reduced significantly after PC; these were mostly concentrated in the Rift Valley. While the mechanistic approach was less prone to this than the Markov approach, both models underestimated prevalence in these areas because of the limited impact of PC on prevalence compared with the rest of the study area where PC was observed to significantly reduce prevalence. This observed variation in PC effectiveness within the study area may be a result of measurement error in the PC coverage data or in the baseline and impact prevalence survey data. Interestingly, this was not the case for hookworm spp., with prevalence consistently declining following PC in all IUs, and consequently both models predicted prevalence at impact accurately. The local scaling approach used to project proxy prevalence surfaces at impact by scaling baseline prevalence predictions from the geostatistical model by IU-level projections assumed that the spatial distribution of predictions at impact was conditional on the spatial distribution at baseline. We found that, for our case study, this was a good approximation, but in future applications it will perform less well in areas where there are abrupt changes in the spatial variation in prevalence within an IU between baseline and impact—for example, due to high spatial variation in PC uptake. Additionally, future applications in geographical regions with PC programs for lymphatic filariasis and onchocerciasis should also account for the anthelminthic impact of the drugs delivered through these programs (albendazole, ivermectin, and diethylcarbamazine) to correctly account for the effect of PC in these areas and ensure good prediction performance.

In the context of survey design, and in statistical design more generally, any sample size calculation must be made on the basis of assumptions that represent a best guess at the true state of the natural process under investigation. The better the guess, the more likely the chosen design will deliver the required precision while avoiding wasteful oversampling, but this can never be guaranteed. Our projected prevalence surfaces at impact were reasonably well calibrated against geostatistical predictions using the impact survey data, albeit with considerable uncertainty and with some exceptions, most notably with respect to some species in the Rift Valley region where the Markov approach performed particularly badly. Despite these challenges, our simulation study demonstrated that the projected surfaces from the mechanistic approach and, to a lesser degree, from the Markov approach were highly informative for guiding survey design as measured by the proportion of correctly classified IUs. Given the budget constraints that apply in regions where STHs are prevalent, both the design of an IAS and the subsequent analysis of IAS data should be conducted as efficiently as possible. Our simulation study only considered spatially random sampling, but further improvements in the efficiency of a survey design can sometimes be achieved by spatially regulated sampling, as has been shown previously [6].

For the design of future impact surveys in other geographical areas, the Markov model is limited by its reliance on midterm data to estimate the effect of PC. In the absence of midterm survey data, the Markov model could still be applied using parameters for PC efficacy estimated from this Kenya case study. However, in this case, the mechanistic model, which does not require midterm data, is likely to perform better and therefore to be more generalizable to other geographical areas because it explicitly models the interaction between PC and STH transmission dynamics.

This study demonstrated that the mechanistic approach more accurately projected prevalence at impact and provided more accurate information for guiding survey design. The usefulness of the 2 approaches for projecting and survey design considered in this study is not confined to STHs. The Markov model is not disease specific and can be applied directly to other NTDs that are controlled with PC. The mechanistic model used here is an STH-specific transmission model and would need to be replaced with a validated transmission model for any other NTD of interest. Our results suggest that, if a validated transmission model is available, it should be used to guide survey design. For both approaches, environmental explanatory variables that are known to be predictors of prevalence for the NTD of interest should be included in the geostatistical models used for predicting prevalence at baseline.

## Supplementary Data

Supplementary materials are available at *Clinical Infectious Diseases* online. Consisting of data provided by the authors to benefit the reader, the posted materials are not copyedited and are the sole responsibility of the authors, so questions or comments should be addressed to the corresponding author.

## Supplementary Material

ciae022_Supplementary_Data

## Notes

***Financial support.*** This work was supported by the Bill and Melinda Gates Foundation via grant number INV-030046 to the NTD Modelling Consortium: Informing Program Decision-Making.

***Supplement sponsorship.*** This article appears as part of the supplement “New Tools and Nuanced Interventions to Accelerate Achievement of 2030 Roadmap for Neglected Tropical Diseases,” sponsored by funding of Professor T. Déirdre Hollingsworth's research by the Li Ka Shing Foundation at the Big Data Institute, Li Ka Shing Centre for Health Information and Discovery, University of Oxford; and funding of the NTD Modelling Consortium by the Bill & Melinda Gates Foundation (INV-030046).
