# Supplementary material for: A Comparison of Markov and Mechanistic Models for Soil-Transmitted Helminth Prevalence Projections in the Context of Survey Design
Source: Clin Infect Dis. 2024 Apr 25;78(Suppl 2):S146–52. doi: 10.1093/cid/ciae022 (PMC11045013; doi:10.1093/cid/ciae022)
Supplement: ciae022_Supplementary_Data [file ciae022_supplementary_data.zip › SF2.pdf]

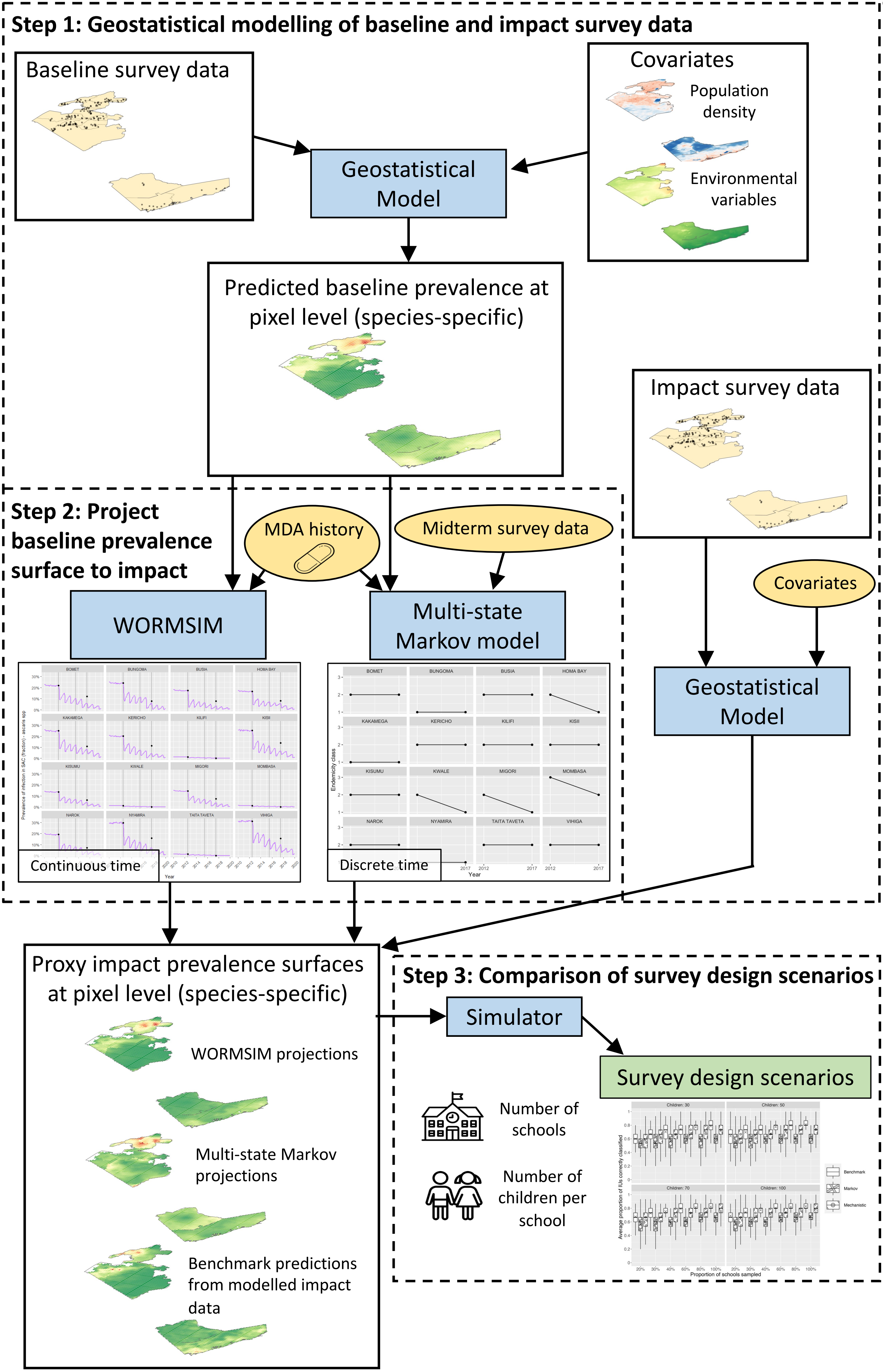

Step 1: Geostatistical modelling of baseline and impact survey data
Baseline survey data
Covariates
Population density
Environmental variables
Geostatistical Model
Predicted baseline prevalence at pixel level (species-specific)
Impact survey data
Step 2: Project baseline prevalence surface to impact
MDA history
Midterm survey data
Covariates
WORMSIM
Multi-state Markov model
Geostatistical Model
Continuous time
Discrete time
Proxy impact prevalence surfaces at pixel level (species-specific)
WORMSIM projections
Multi-state Markov projections
Benchmark predictions from modelled impact data
Step 3: Comparison of survey design scenarios
Simulator
Survey design scenarios
Number of schools
Number of children per school
