# Supplementary material for: A Comparison of Markov and Mechanistic Models for Soil-Transmitted Helminth Prevalence Projections in the Context of Survey Design
Source: Clin Infect Dis. 2024 Apr 25;78(Suppl 2):S146–52. doi: 10.1093/cid/ciae022 (PMC11045013; doi:10.1093/cid/ciae022)

**Supplementary Material**

## Supplementary Section 1. Geostatistical modelling framework

**S1.1 The geostatistical model**

We denote by $P\left( x \right)$ the prevalence of STH at location $x$. Our model for the variation in $P\left( x \right)$throughout the region of interest is that

$log\left( \frac{P\left( x \right)}{\left( 1-P\left( x \right) \right)} \right)=d\left( x \right)'\beta+S\left( x \right)+Z.$ Eqn (1)

In Equation (1), $d\left( x \right)$ is a vector of covariates associated with regression coefficients β. This component of the model accounts for variation in prevalence that can be explained by measured characteristics of the location $x$. The terms $S\left( x \right)$ and $Z$ account for any remaining variation that cannot be explained by measured characteristics of $x$. The term $S\left( x \right)$is a spatially correlated Gaussian process with mean zero and covariance structure

$Cov\left( S\left( x \right),S\left( x' \right) \right)=\sigma^{2}\rho\left( u;\theta\right),$

where $u=\|x-x'\|$ is the Euclidean distance between $x$ and $x^{'},$ $\sigma^{2}$ is the variance and

$\rho\left( u;\phi\right)=expexp \left( -\frac{u}{\phi} \right)$

is the correlation between $S\left( x \right)$ and $S\left( x' \right)$. The term $Z$ in Equation (1) is a Normally distributed random variable with zero mean and variance $\tau^{2}$ that varies independently between locations; it accounts for variation in unmeasured characteristics of the sampled individuals that affect their personal exposure to STH.

We denote by $x_{1},\ldots,x_{n}$ the set of sampled locations. Conditional on $P\left( x_{i} \right)$, the numbers $Y_{i}$ of individuals who test positive out of $m_{i}$ sampled individuals at $x_{i}$ are independent binomially distributed random variables, with binomial probabilities $P\left( x_{i} \right)$ and denominators $m_{i}$.

**S1.2 Parameter estimation**

We carry out parameter estimation using Monte Carlo Maximum Likelihood (MCML), implemented in PrevMap, an R package for analysing prevalence data, freely available from the Comprehensive R Archive Network (*www.r-project.org*).

Let $\eta_{i}=log\left( \frac{P\left( x \right)}{\left( 1-P\left( x \right) \right)} \right)$. The joint conditional density of $Y=Y_{1},\ldots,Y_{n}$is

$f\left( y|\eta\right)=\prod_{i=1}^{n} f\left( y_{i}|\eta_{i} \right)$.

The likelihood function for the set of model parameters $\psi$ is obtained by integrating out the random components$S\left( x_{i} \right)$and$Z_{i}$ from $\eta_{i}$, hence

$L\left( \psi\right)=\int_{R^{n}} f\left( y|\eta\right) f\left( \eta;\psi\right)d\eta$ Eqn (2)

where$f\left( \eta;\psi\right)$is a multivariate Normal density.

To approximate the integral in Equation 2 we use a Markov Chain Monte Carlo (MCMC) algorithm to generate a sample $\eta_{\left( 1 \right)},...,\eta_{\left( N \right)}$ from the conditional distribution of $\eta$ given $y$ and approximate the likelihood as

$$L\left( \psi\right)\propto L_{N}\left( \psi\right)=\frac{1}{N}\frac{\sum_{j=1}^{N} f\left( \eta_{\left( j \right)};\psi\right)}{f\left( \eta_{\left( j \right)};\psi_{0} \right)},$$

Where $\psi_{0}$ is our best guess for the initial parameter values.

**S1.3 Prediction**

Here, we use plug-in prediction, meaning that we use the Monte Carlo maximum likelihood parameter estimate $\psi^$ in place of the unknown $\psi$.

Our goal is to predict prevalence throughout the region of interest, $A$. We approximate this by a regular grid of points $x_{n+1},...,x_{n+q}$ that cover $A$. Our predictive target is the set of values

$\eta_{n+i}=d\left( x_{n+i} \right)'\beta+S\left( x_{n+i} \right)$ Eqn (3)

Note that Equation 3 excludes the term $Z$ in Equation 1, which relates to characteristics of the sampled individuals at a location rather than of the location itself.

The *predictive distribution* of $\eta^{*}=\left( \eta_{n+1},...,\eta_{n+q} \right)$ is its conditional distribution given $y$,

$$f\left( \eta^{*}|y \right)=\int_{R^{n}} f\left( \eta^{*}|\eta\right)f\left( \eta|y \right)d\eta,$$

where we have used the fact that $\eta^{*}$ and $y$ are conditionally independent given $\eta$. It follows that to generate a sample from the predictive distribution of $\eta^{*}$ we first sample from $f\left( \eta|y \right)$ and then from$f\left( \eta^{*}|\eta\right)$. A sample from the joint predictive distribution of prevalence throughout $A$ follows by direct transformation, using the formula

$$P\left( x_{n+i} \right)=\frac{exp\left( \eta_{n+1}^{*} \right)}{1+exp\left( \eta_{n+1}^{*} \right)}.$$

Our point prediction of prevalence at any location $x$ is the mean of the sampled values of $P\left( x \right)$. The predictive probability that prevalence lies within any stated range, say $c$ to $d$, is the proportion of sampled values that lie between $c$and$d$. The predictive distribution of prevalence at the implementation unit level, $P_{IU}$, is computed as a population-weighted average of the pixel level prevalence $P\left( x \right)$, hence

$$P_{IU}=\frac{\int P\left( x \right)w\left( x \right)dx}{\int w\left( x \right)dx}$$

where $w\left( x \right)$ is an estimate of the population at location $x$ obtained from WorldPop (*https://www.worldpop.org/*) and the integral is over the whole of the IU.

**Supplementary Section 2. Description of WORMSIM**

WORMSIM simulates the life histories of individual humans and individual worms within a closed human population. The population-level age- and sex-distribution are based on pre-specified fertility rates and life tables. A comparison of the population distribution for WORMSIM and the Kenyan population is provided in (see **Supplementary Figure 2.1** below).


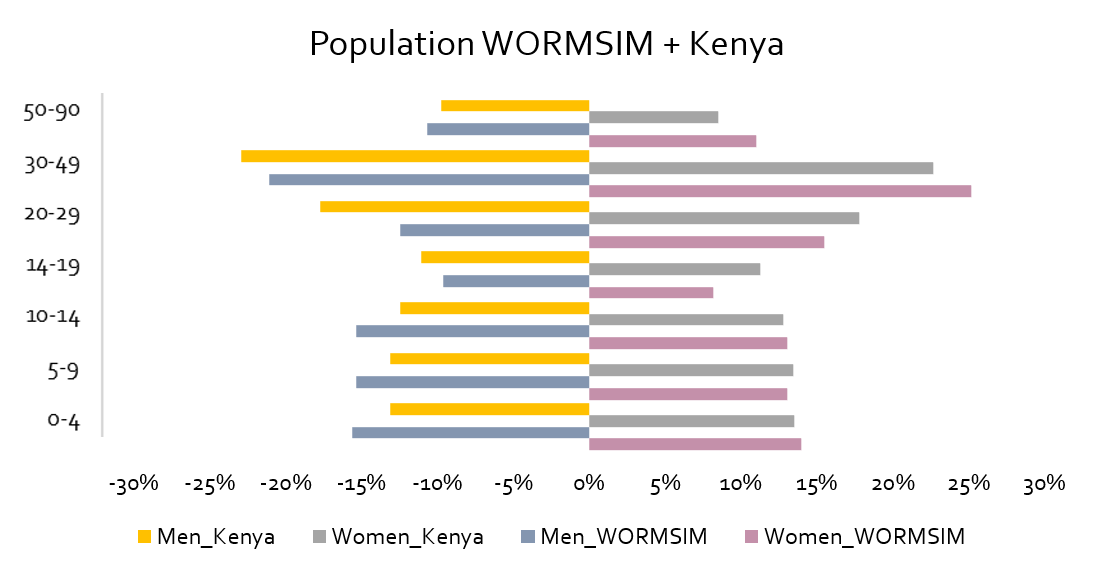


**Supplementary Figure 2.1.** Population age pyramid by sex for WORMSIM population (left side of the chart) and Kenyan population (right side of the chart).

The prevalence and intensity distribution of worm infections is determined by model parameters for the transmission rate (overall rate and differences by age) and level of exposure heterogeneity within a population. For this study, we assume standard level of exposure heterogeneity for each species (gamma distribution with shape parameter *k* (13)), and we adapted the overall transmission rate for each IU to replicate IU-level baseline prevalences of infection as estimated from the NSBDP data. The overall transmission rate was calibrated following two different assumptions: transmission increasing with age versus transmission being independent of age (13,15). However, in another widely applied individual-based stochastic transmission model by Imperial College London (ICL) (15), the modellers assume that age-dependent contribution to infection is proportional to age-dependent exposure.

For *T. trichiura* and *A. lumbricoides*, we explored two different values for (species-specific) exposure heterogeneity (= p1), to reflect different potential exposure and transmission dynamics. Simulating stable transmission dynamics with very low overall prevalence levels is known to be challenging with WORMSIM (13,16). Therefore, if baseline prevalence levels were lower than a certain value, we ran the model simulations for a higher cut-off prevalence value (for hookworm *spp.* 20%; for *T. trichiura* 30%; and for *A. lumbricoides* 25%) and scaled the predicted prevalence values back to the measured lower baseline prevalence.

The IU-level baseline prevalence levels to which WORMSIM was calibrated were estimated for each IU by weighting the baseline geostatistical model prevalence prediction surface by population density and calculating two IU-level summary statistics: mean prevalence and prevalence tertiles. Prevalence tertiles were created by taking the grid-level population weighted-prevalence divided into three ordered groups. WORMSIM further simulates the impact of PC on STH infection levels, assuming that treatment kills a proportion of adult worms (efficacy varying by species and drug as in previous studies (13)) and accounting for the proportion of the population that takes up PC. For this study, we adopt coverage levels directly from the data, assuming mixed compliance. For simulating infection with hookworm *spp.*, we explored two different functions for infection exposure by age, further illustrated in a previous comparison study (17). In WORMSIM we originally assume that the contribution and exposure to transmission increases with age up to the age of ten, after which the practice of defecation is assumed to remain similar regardless of age. A similar pattern is applied to *T. trichiura* and *A. lumbricoides*.

For each species, we simulated different model scenarios for different levels of exposure heterogeneity, age-dependent exposure patterns and baseline (**Supplementary Table 2.1** and **Supplementary Figures 9-11**), and selected the most appropriate scenario based on the expected effectivity of school-based PC in SAC as reported in the scientific literature (16). For all three species, we simulated prevalence levels over time based on each of the aforementioned prevalence summary statistics i) IU-level mean population-weighted prevalence and ii) IU-level population-weighted prevalence tertiles to account for real-life spatial heterogeneity in prevalence that might influence infection dynamics.

To generate a proxy impact surface, local scaling of the predicted baseline prevalence surface was then conducted using the prevalence simulations at impact generated by WORMSIM for each IU following the methodology described in ‘Local scaling to generate a proxy impact surface’.

The predicted *A. lumbricoides* prevalence using four different model scenarios is provided in **Supplementary Figure 10**. The predicted prevalence from the final model (model #1) and measured baseline and impact prevalence levels are displayed in **Supplementary** **Figure 11**. The predicted *T. trichiura* prevalence using 4 different model scenarios is provided in **Supplementary Figure 12**. The predicted prevalence from the final model (model #1) and measured baseline and impact prevalence levels are displayed in **Supplementary Figure 13**. The predicted hookworm spp. prevalence using different model scenarios is provided in **Supplementary Figure 14**. The predicted prevalence from the final model (model #1) and measured baseline and impact prevalence levels are displayed in **Supplementary Figure 15**.

**Supplementary Table 2.1.** Overview of explored WORMSIM models shown for each STH species.

|  |  | ***A. lumbricoides*** | ***T. trichiura*** | **Hookworm *spp.*** |
| --- | --- | --- | --- | --- |
| **Model 1** | Exposure heterogeneity | High exposure (p1 = 0.8) | High exposure (p1 = 0.35) | High exposure (p1 = 0.35) |
|  | Age-exposure function | Assumption of transmission increasing with age (EMC model) | Assumption of transmission increasing with age (EMC model) | Assumption of transmission being independent of age (ICL model) |
|  | Baseline prevalence | Based on tertiles | Based on tertiles | Based on tertiles |
| **Model 2** | Exposure heterogeneity | High exposure (p1 = 0.8) | High exposure (p1 = 0.35) | High exposure (p1 = 0.35) |
|  | Age-exposure function | Assumption of transmission increasing with age (EMC model) | Assumption of transmission increasing with age (EMC model) | Assumption of transmission being independent of age (ICL model) |
|  | Baseline prevalence | Overall mean | Overall mean | Overall mean |
| **Model 3** | Exposure heterogeneity | Low exposure (p1 = 0.4) | Low exposure (p1 = 0.2) | High exposure (p1 = 0.35) |
|  | Age-exposure function | Assumption of transmission increasing with age (EMC model) | Assumption of transmission increasing with age (EMC model) | Assumption of transmission increasing with age (EMC model) |
|  | Baseline prevalence | Based on tertiles | Based on tertiles | Based on tertiles |
| **Model 4** | Exposure heterogeneity | Low exposure (p1 = 0.4) | Low exposure (p1 = 0.2) | High exposure (p1 = 0.35) |
|  | Age-exposure function | Assumption of transmission increasing with age (EMC model) | Assumption of transmission increasing with age (EMC model) | Assumption of transmission increasing with age (EMC model) |
|  | Baseline prevalence | Overall mean | Overall mean | Overall mean |

**Supplementary Figure 1.** Preventive chemotherapy (PC) coverage data between baseline and impact surveys for the 16 IUs in Southwest Kenya. Data was obtained through Evidence Action.


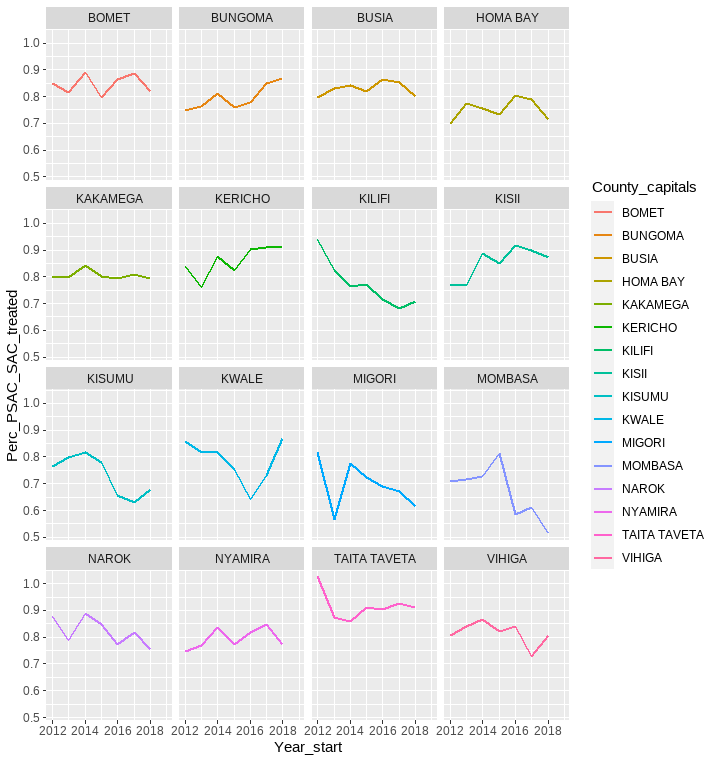


**Supplementary Figure 2.** Overview of the study modelling process.

**Supplementary Figure 3.** Generalized Additive Model (GAM) and linear model dependence plots for the log-odds of *A. lumbricoides* prevalence at baseline plotted against the continuous environmental covariates considered in this analysis (data are plotted as points and shaded areas correspond to 95% confidence intervals): EVI (Enhanced Vegetation Index), mean daytime LST (land surface temperature), mean night-time LST, soil acidity.


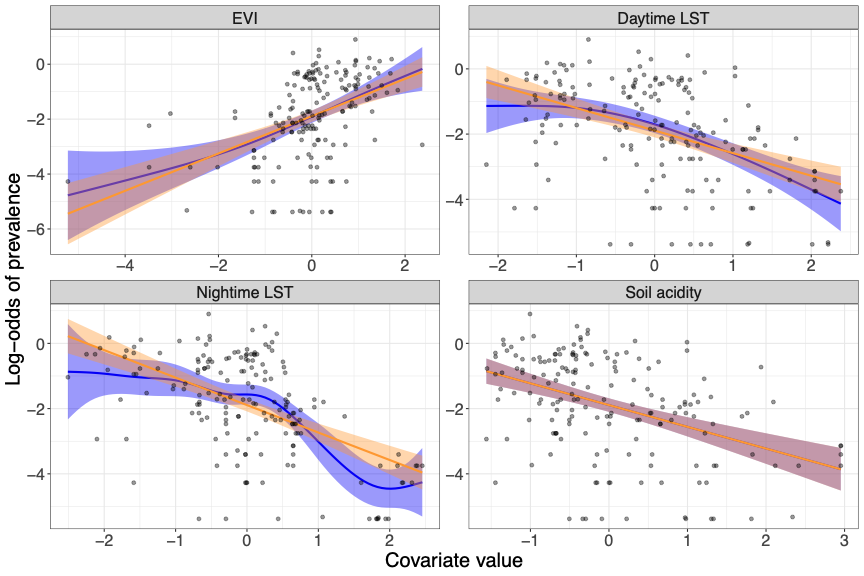


**Supplementary Figure 4.** Generalized Additive Model (GAM) and linear model dependence plots for the log-odds of *T. trichiura* prevalence at baseline plotted against the continuous environmental covariates considered in this analysis (shaded areas correspond to 95% confidence intervals): EVI (Enhanced Vegetation Index), mean daytime LST (land surface temperature), mean nighttime LST, soil acidity.


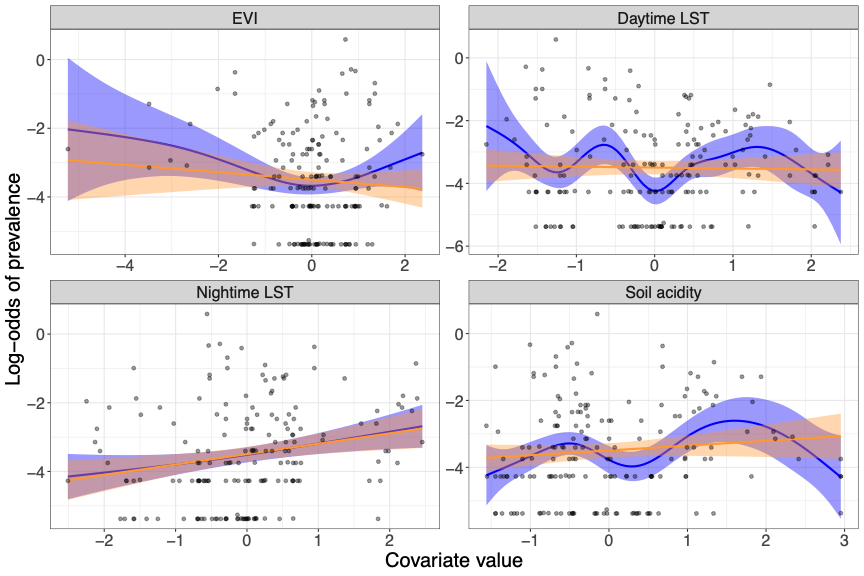


**Supplementary Figure 5.** Generalized Additive Model (GAM) and linear model dependence plots for the log-odds of hookworm *spp.* prevalence at baseline plotted against the continuous environmental covariates considered in this analysis (shaded areas correspond to 95% confidence intervals): EVI (Enhanced Vegetation Index), mean daytime LST (land surface temperature), mean night-time LST, soil acidity.


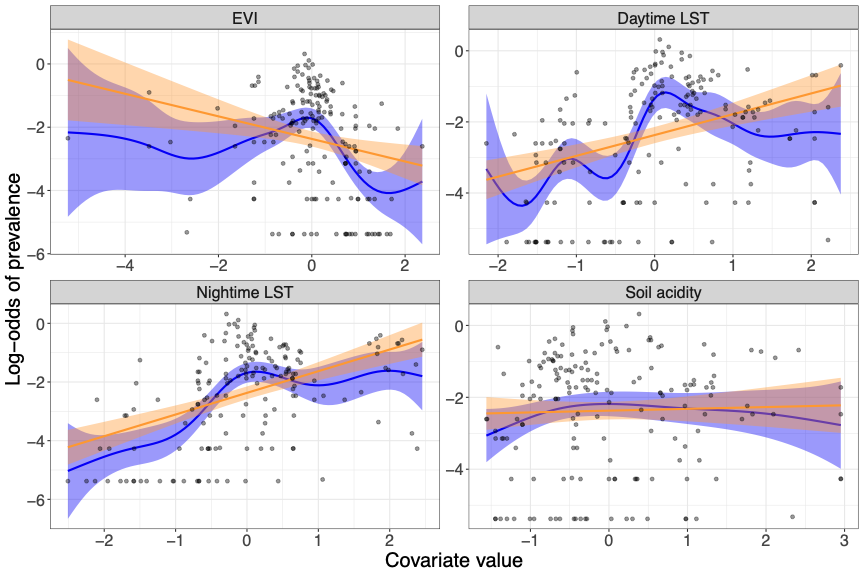


**Supplementary Table 1.** Monte Carlo maximum likelihood estimates and corresponding 95% confidence intervals for the baseline geostatistical model (continuous covariates were standardised).

|  | ***A. lumbricoides*** | ***T. trichiura*** | **Hookworm *spp.*** |
| --- | --- | --- | --- |
| EVI | 0.265 (-0.105, 0.635) | 0.018 (-0.392, 0.428) | 0.279 (-0.070, 0.628) |
| LST (day) | -0.101 (-0.502, 0.299) | -0.161 (-0.683, 0.362) | 0.360 (-0.070, 0.790) |
| LST (night) | -0.291 (-0.794, 0.213) | 0.252 (-0.658, 1.162) | 0.748 (0.126, 1.371) |
| Soil acidity | 0.013 (-0.401, 0.426) | -0.049 (-0.589, 0.491 | -0.286 (-0.717, 0.145 |
| Region - Coast (ref) | - | - | - |
| Region - Nyanza | 2.346 (1.189, 3.502) | -1.191 (-4.107, 1.726) | 1.126 (-0.403, 2.656) |
| Region - Rift valley | 2.137 (0.800, 3.475) | 1.446 (-1.312, 4.203) | -0.435 (-2.090, 1.220) |
| Region - Western | 2.842 (1.755, 3.929) | -0.561 (-3.165, 2.043) | 1.462 (-0.042, 2.965) |
| 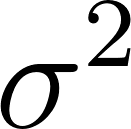 | 0.726 (0.395, 1.333) | 1.967 (0.742, 5.210) | 0.865 (0.374, 1.998) |
| 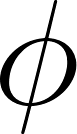 (km) | 8.517 (3.598, 20.161) | 53.296 (14.070, 201.885) | 24.356 (6.167, 96.197) |
| 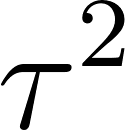 | 0.372 (0.130, 2.024) | 0.749 (0.120, 1.206) | 0.550 (0.209, 1.936) |

**Supplementary Figure 6.** Generalized Additive Model (GAM) and linear model dependence plots for the log-odds of *A. lumbricoides* prevalence at impact plotted against the continuous environmental covariates considered in this analysis (data are plotted as points and shaded areas correspond to 95% confidence intervals): EVI (Enhanced Vegetation Index), mean daytime LST (land surface temperature), mean night-time LST, soil acidity.


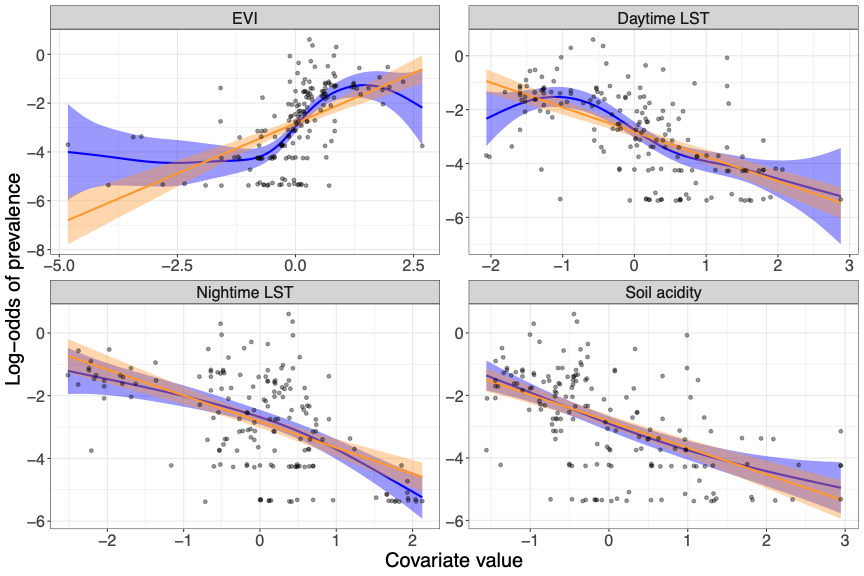


**Supplementary Figure 7.** Generalized Additive Model (GAM) and linear model dependence plots for the log-odds of *T. trichiura* prevalence at impact plotted against the continuous environmental covariates considered in this analysis (data are plotted as points and shaded areas correspond to 95% confidence intervals): EVI (Enhanced Vegetation Index), mean daytime LST (land surface temperature), mean night-time LST, soil acidity.


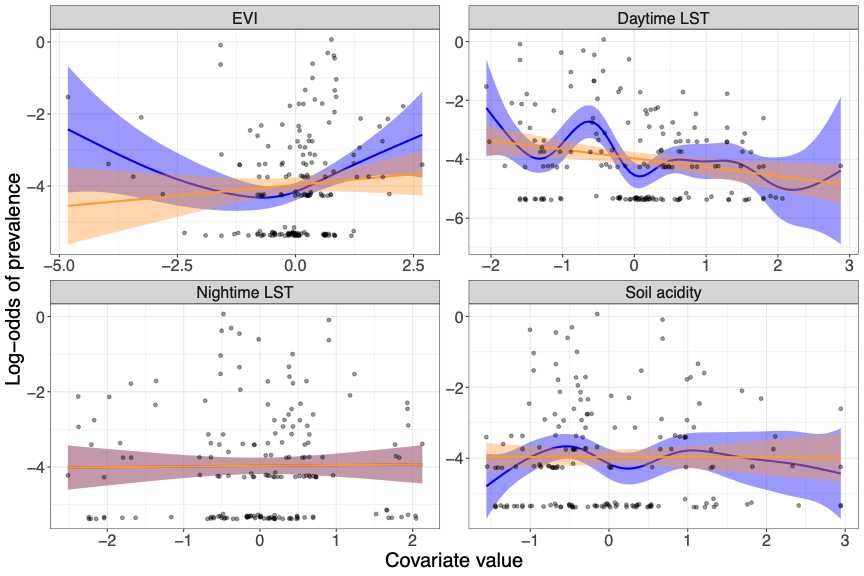


**Supplementary Figure 8.** Generalized Additive Model (GAM) and linear model dependence plots for the log-odds of hookworm *spp.* prevalence at impact plotted against the continuous environmental covariates considered in this analysis (data are plotted as points and shaded areas correspond to 95% confidence intervals): EVI (Enhanced Vegetation Index), mean daytime LST (land surface temperature), mean night-time LST, soil acidity.


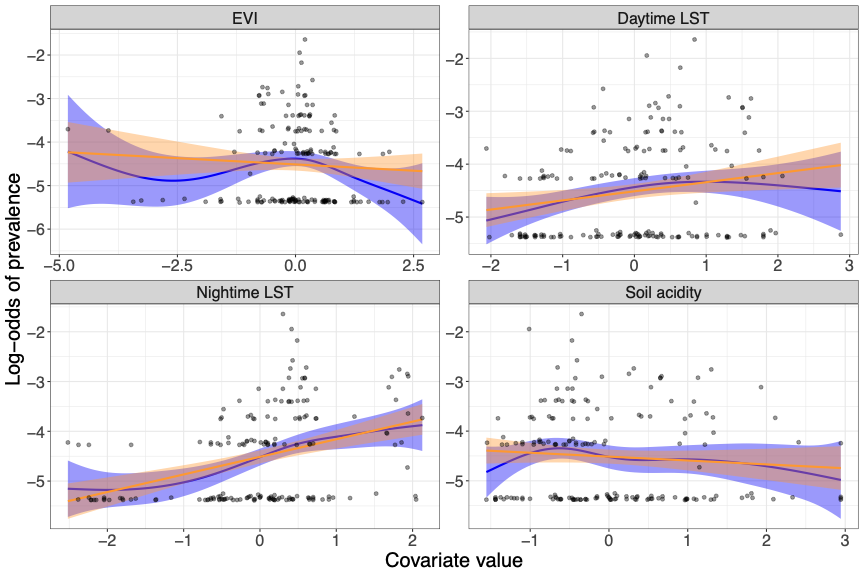


**Supplementary Table 2.** Monte Carlo maximum likelihood estimates and corresponding 95% confidence intervals for the baseline geostatistical model (continuous covariates were standardised).

|  | ***A. lumbricoides*** | ***T. trichiura*** | **Hookworm *spp.*** |
| --- | --- | --- | --- |
| EVI | 0.565 (0.158, 0.972) | 0.347 (-0.069, 0.763) | 0.290 (0.046, 0.533) |
| LST (day) | -0.642 (-1.069, -0.215) | -0.746 (-1.321, -0.172) | 0.191 (-0.080, 0.463) |
| LST (night) | 0.386 (-0.132, 0.904) | 1.276 (0.363, 2.188) | 1.328 (0.962, 1.693) |
| Soil acidity | -0.079 (-0.522, 0.364) | 0.090 (-0.483, 0.662) | -0.575 (-0.870, -0.279) |
| Region - Coast (ref) | - | - | - |
| Region - Nyanza | 5.003 (2.941, 7.064) | 1.005 (-1.941, 3.951) | 1.158 (0.337, 1.979) |
| Region - Rift valley | 5.057 (2.971, 7.143) | 4.149 (1.384, 6.914) | 0.750 (-0.240, 1.740) |
| Region - Western | 5.019 (3.029, 7.009) | 0.933 (-1.481, 3.346) | 0.988 (0.268, 1.708) |
| 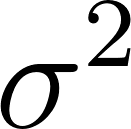 | 0.903 (0.566, 1.439) | 1.708 (0.574, 5.086) | 0.559 (0.389, 0.803) |
| 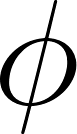 (km) | 6.530 (3.271, 13.040) | 38.231 (8.182, 178.633) | 8.518 (5.063, 14.329) |
| 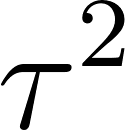 | 0.145 (0.022, 1.171) | 0.602 (0.089, 1.395) | 0.041 (0.013, 0.422) |

**Supplementary Figure 9.** Predicted prevalence for the Markov model for i) *A. lumbricoides*, ii) *Hookworm spp.* And iii) *T. trichiura* among school-aged children (SAC) at 16 IUs in Southwest Kenya, plotted against the benchmark prevalence from the impact survey.


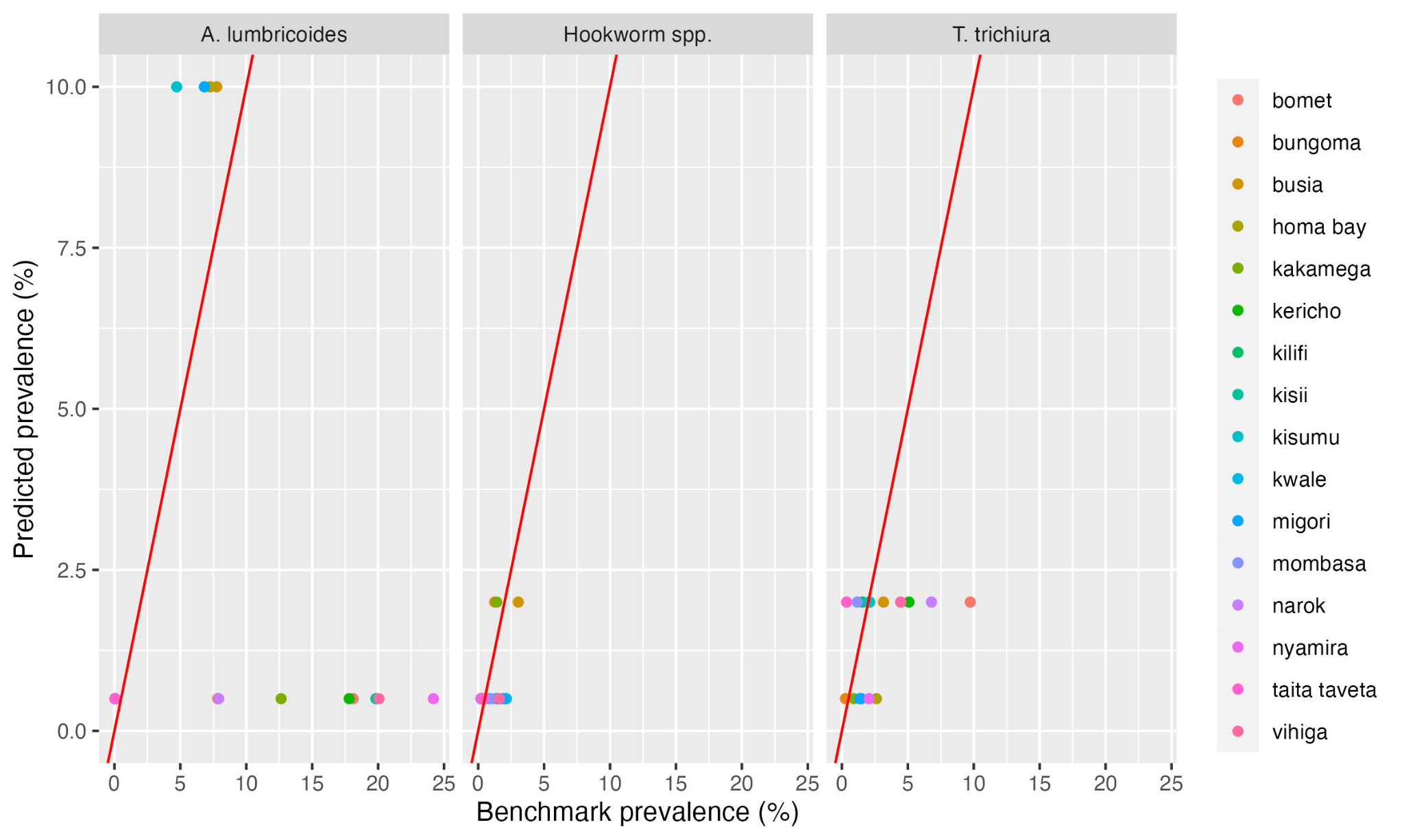


**Supplementary Figure 10.** Predicted *A. lumbricoides* prevalence among school-aged children (SAC) at 16 IUs in Southwest Kenya over time, using 4 different modelling scenarios (described in **Supplementary Table 2**) with WORMSIM. Overall, the models modestly underestimated endline prevalence for the different UIs.


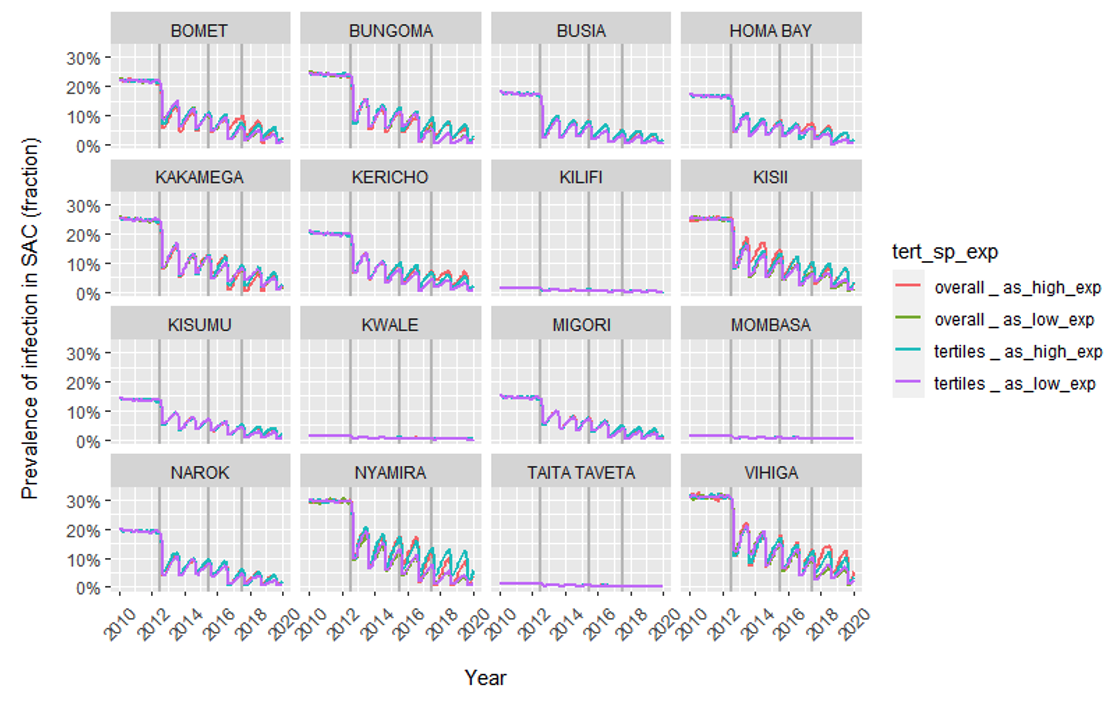


**Supplementary Figure 11.** Predicted *A. lumbricoides* prevalence among school-aged children (SAC) at 16 IUs in Southwest Kenya over time, using WORMSIM. Black points show the measured prevalence levels among SAC at baseline and impact.


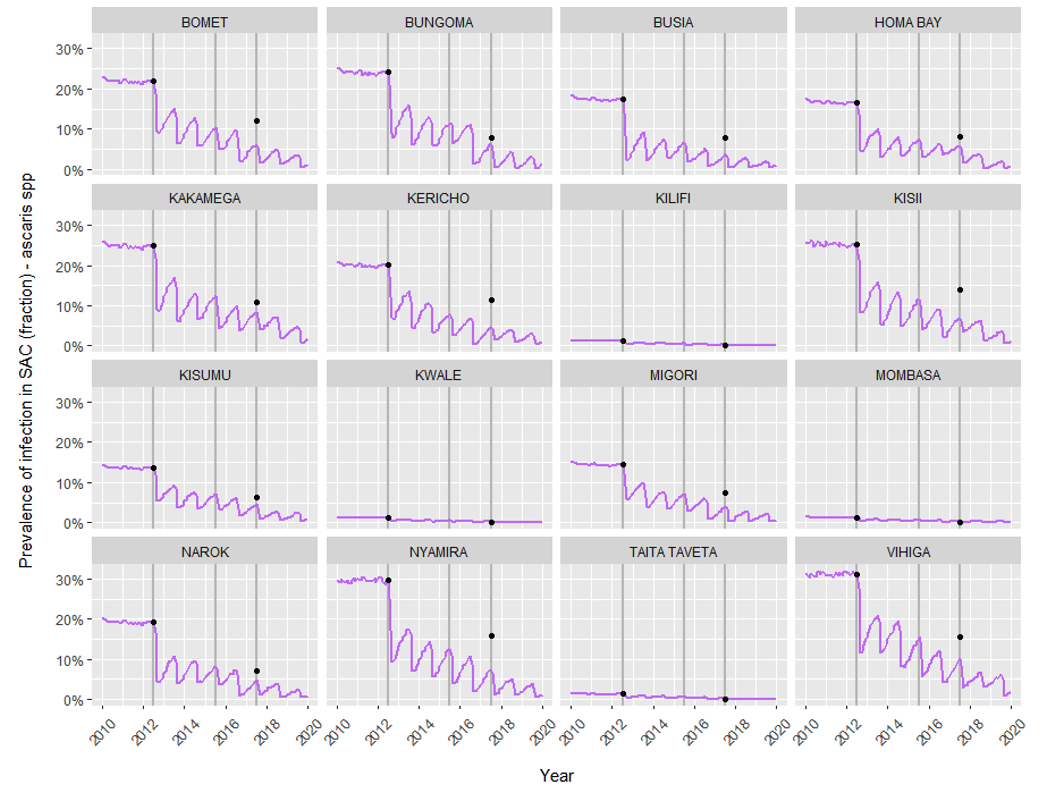


**Supplementary Figure 12.** Predicted *T.* *Trichiura* prevalence among school-aged children (SAC) at 16 IUs in Southwest Kenya over time, using 4 different modelling scenarios (described in **Supplementary Table 2**) with WORMSIM.


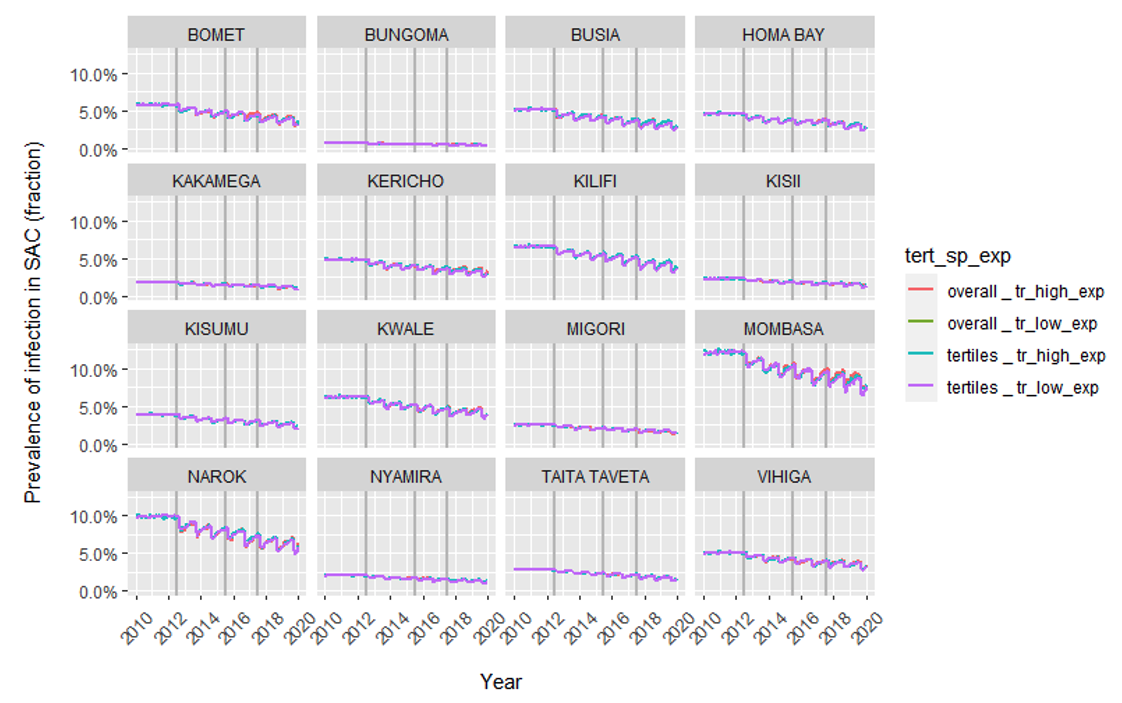


**Supplementary Figure 13.** Predicted *T. trichiura* prevalence among school-aged children (SAC) at 16 IUs in Southwest Kenya over time, using WORMSIM. Black points show the measured prevalence levels among SAC at baseline and impact.


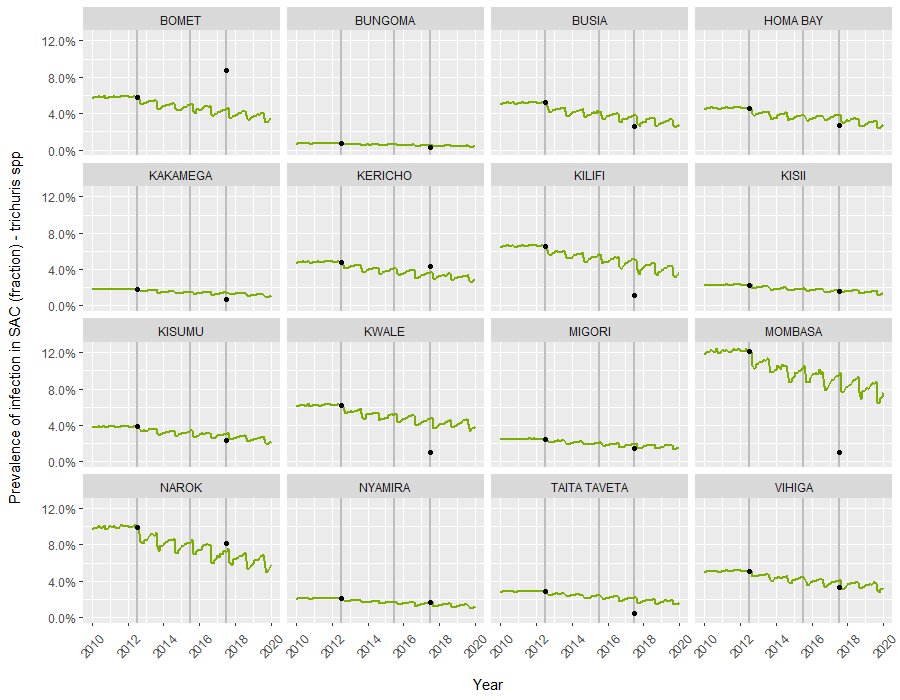


For *T. trichiura*, in contrast to the predictions for *A. lumbricoides*, the model underestimated the prevalence reduction at impact in three IUs.

**Supplementary Figure 14.** Predicted hookworm *spp.* prevalence among school-aged children (SAC) at 16 IUs in Southwest Kenya over time, using 4 different modelling scenarios (described in **Supplementary Table 2**) with WORMSIM.


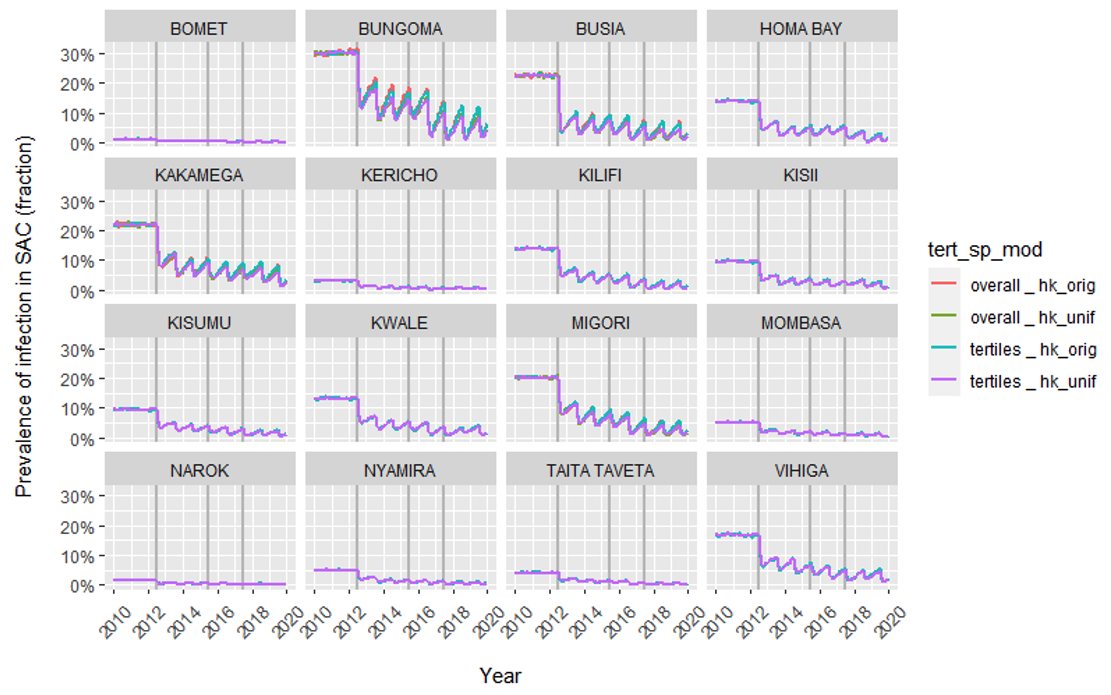


There were no large differences in the predicted impact prevalence based on model #1 and model #3, the two scenarios assume different patterns of age-dependent exposure. Based on the predictions from model #1 (i.e. Erasmus MC exposure function), prevalence in SAC is observed to go up faster after PC. This is due to larger exposure in adults compared to preSAC and SAC, creating a larger ‘pool’ of infected people in older people and hence higher infection pressure, resulting in quicker bounce-backs of prevalence after PC among SAC.

**Supplementary Figure 15.** Predicted hookworm *spp.* prevalence among school-aged children (SAC) at the 16 IUs in Southwest Kenya over time, using WORMSIM. Black points show the measured prevalence levels among SAC at baseline and impact**.**


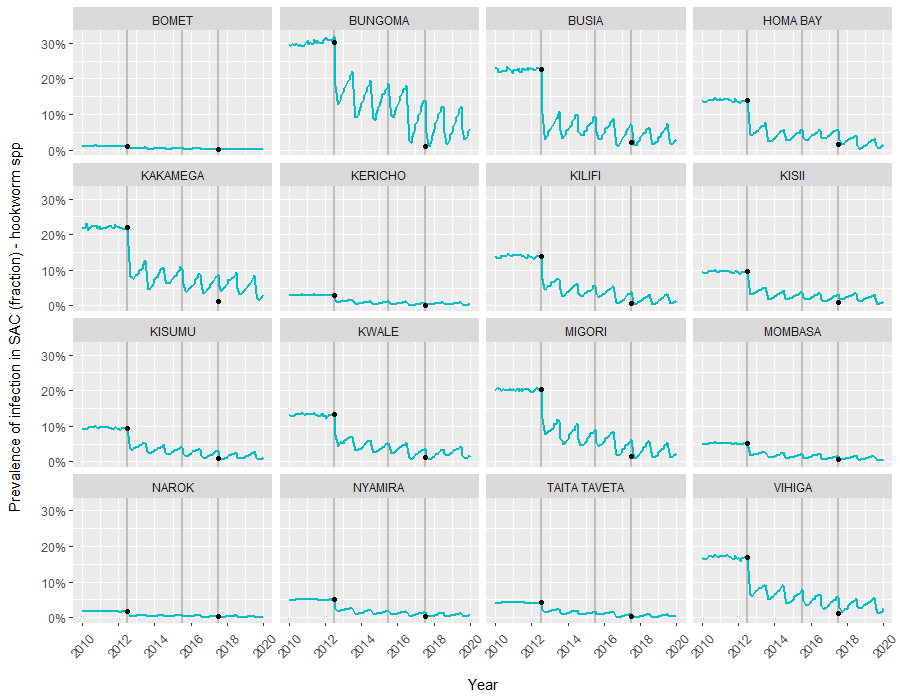


**Supplementary Figure 16.** *A. lumbricoides* prevalence in SAC as predicted by the geostatistical model at baseline and impact, and as projected at impact using the statistical and mechanistic approaches.


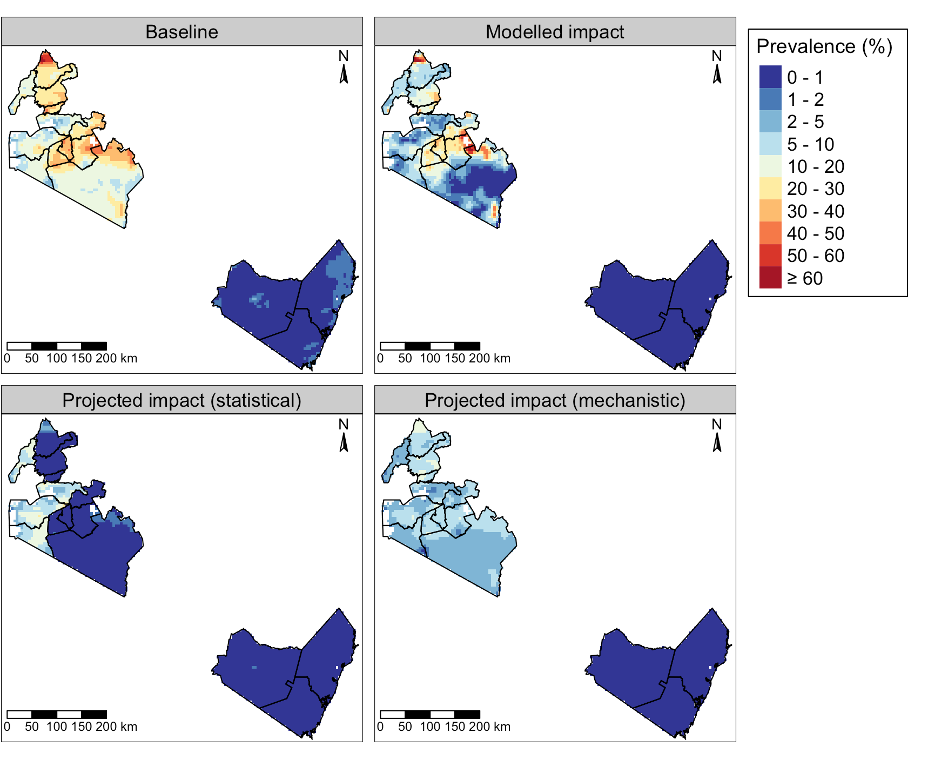


**Supplementary Figure 17.** *T. trichiura* prevalence in SAC as predicted by the geostatistical model at baseline and impact, and as projected at impact using the statistical and mechanistic approaches.


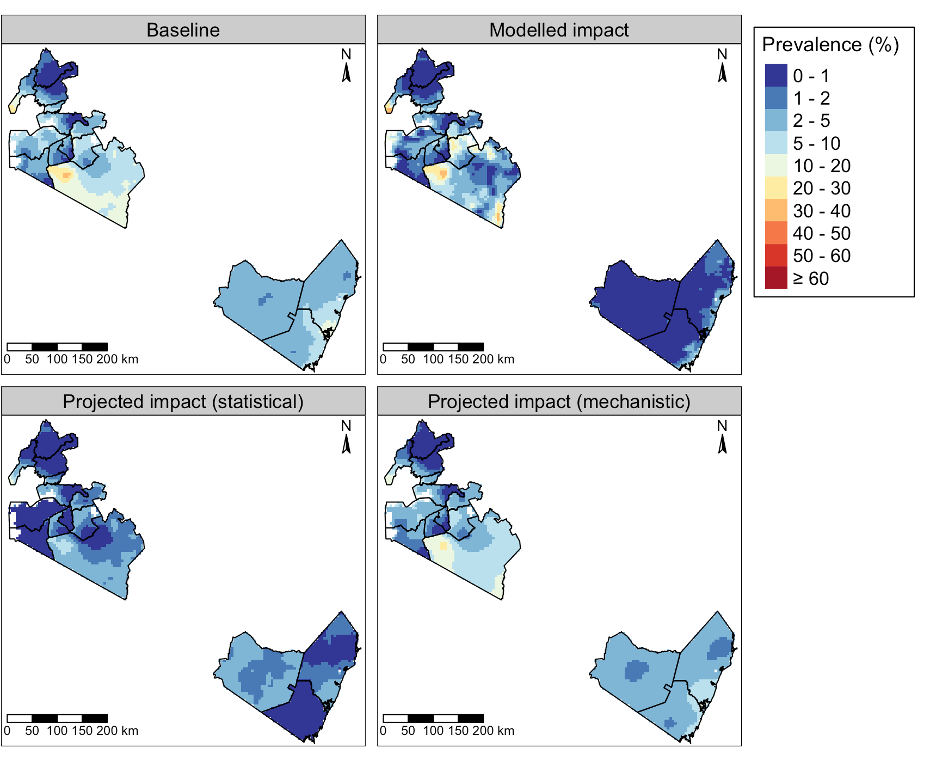


**Supplementary Figure 18.** Hookworm *spp.* prevalence in SAC as predicted by the geostatistical model at baseline and impact, and as projected at impact using the statistical and mechanistic approaches.


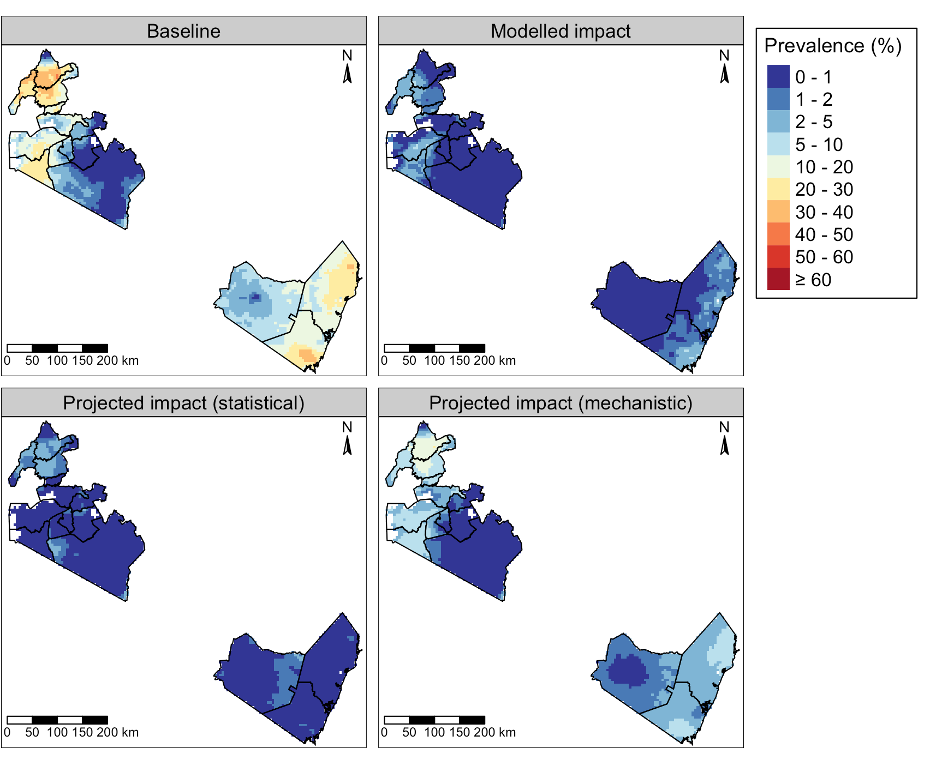

Supplement: ciae022_Supplementary_Data [file ciae022_supplementary_data.zip › Supplementary Material.docx]
