# Supplementary figures and images for: A Comparison of Markov and Mechanistic Models for Soil-Transmitted Helminth Prevalence Projections in the Context of Survey Design
Source: Clin Infect Dis. 2024 Apr 25;78(Suppl 2):S146–52. doi: 10.1093/cid/ciae022 (PMC11045013; doi:10.1093/cid/ciae022)

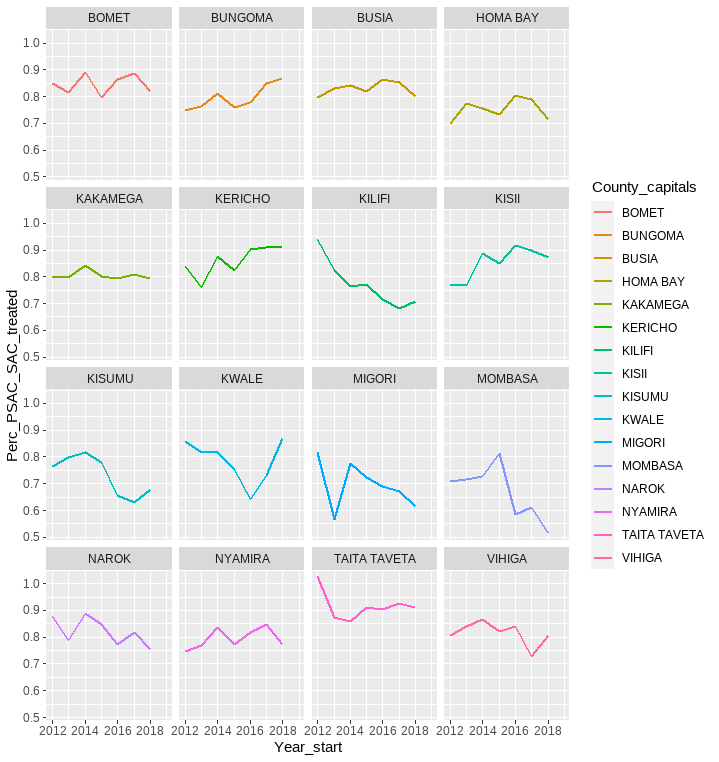

Supplement: ciae022_Supplementary_Data [file ciae022_supplementary_data.zip › SF1.png]

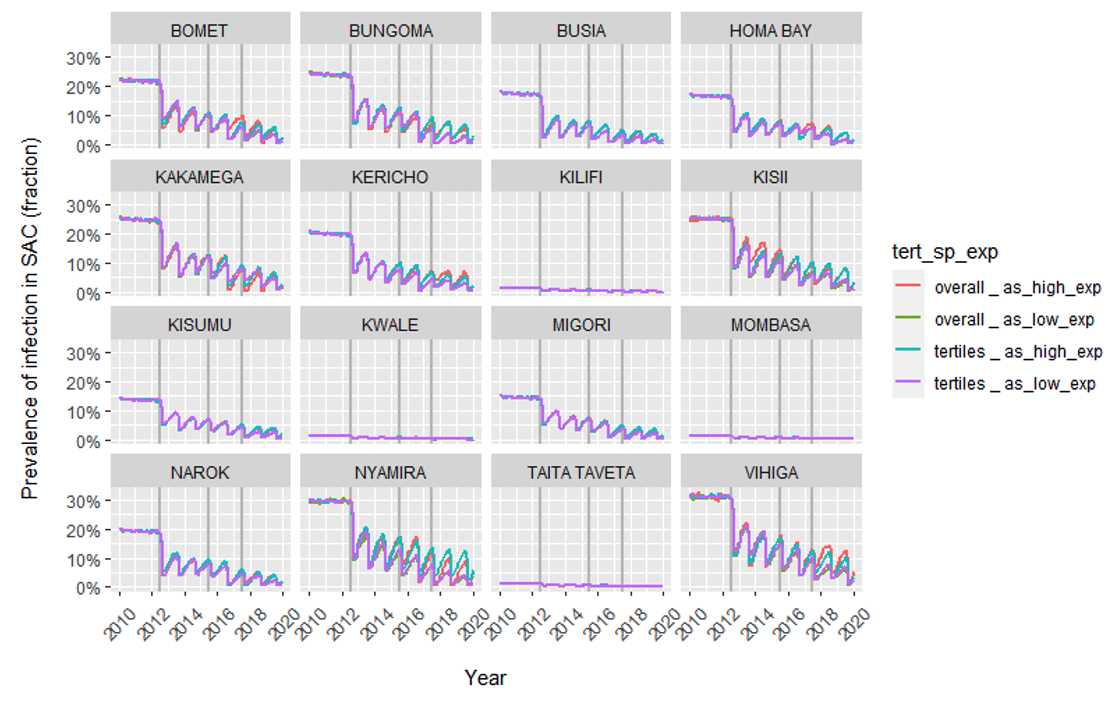

Supplement: ciae022_Supplementary_Data [file ciae022_supplementary_data.zip › SF10.png]

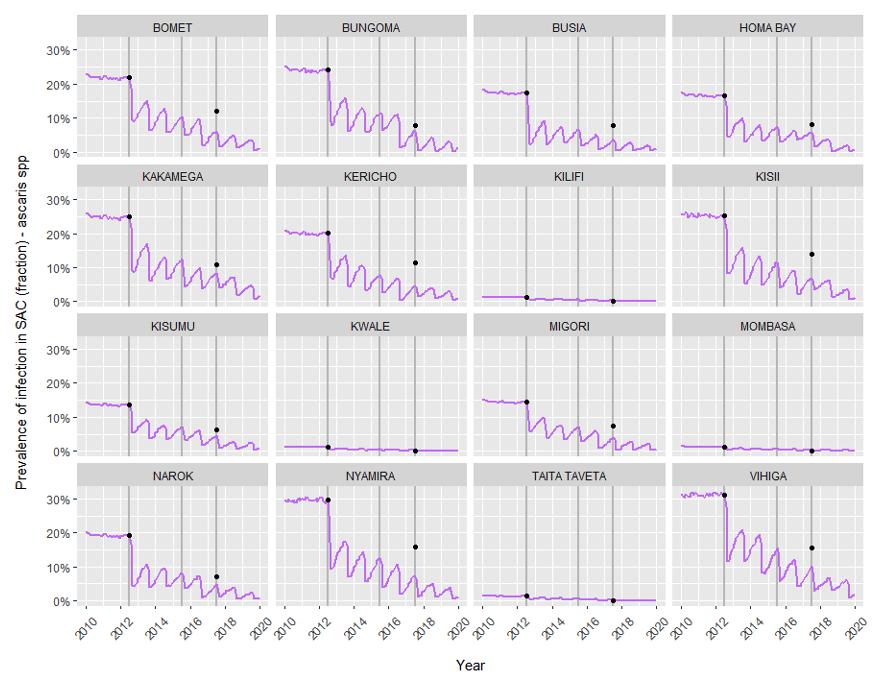

Supplement: ciae022_Supplementary_Data [file ciae022_supplementary_data.zip › SF11.png]

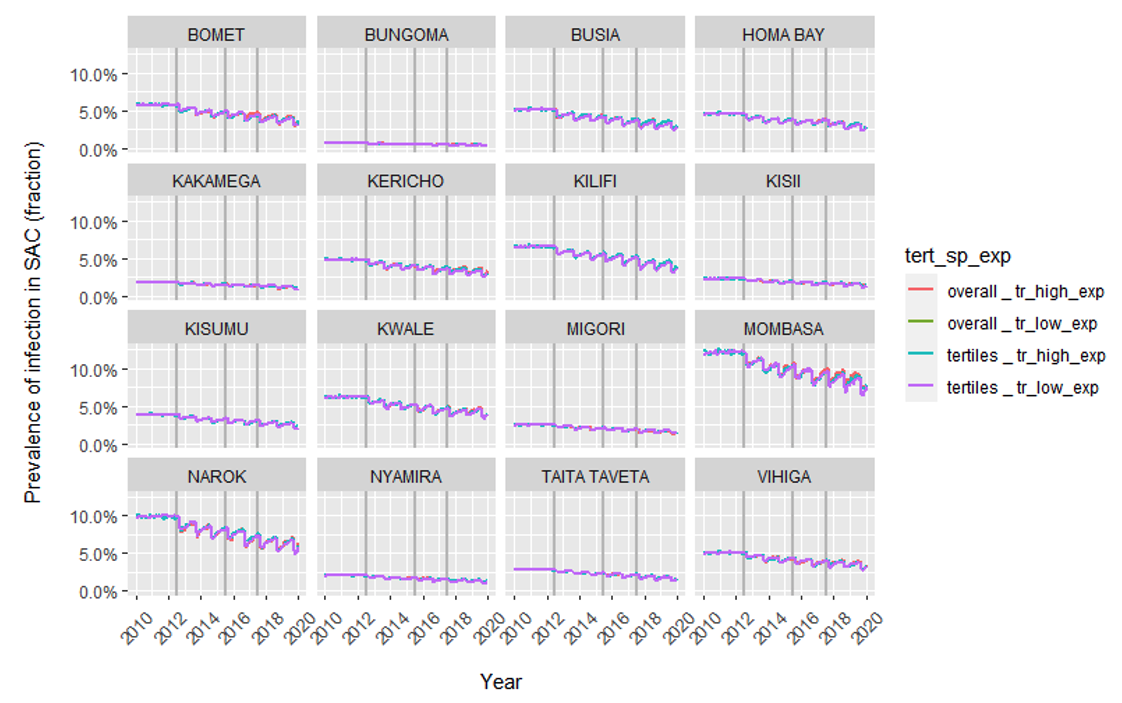

Supplement: ciae022_Supplementary_Data [file ciae022_supplementary_data.zip › SF12.png]

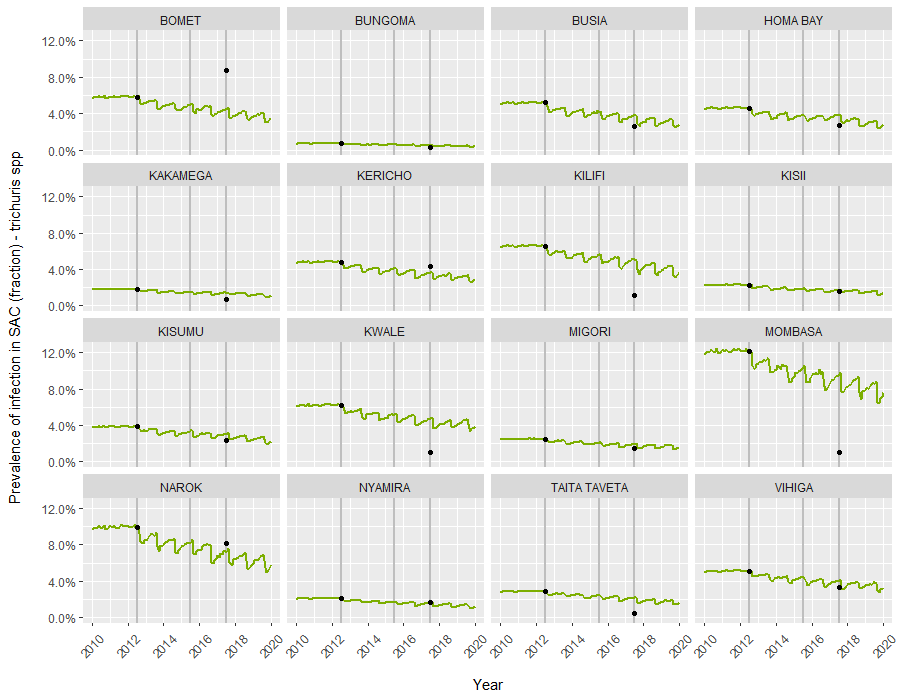

Supplement: ciae022_Supplementary_Data [file ciae022_supplementary_data.zip › SF13.png]

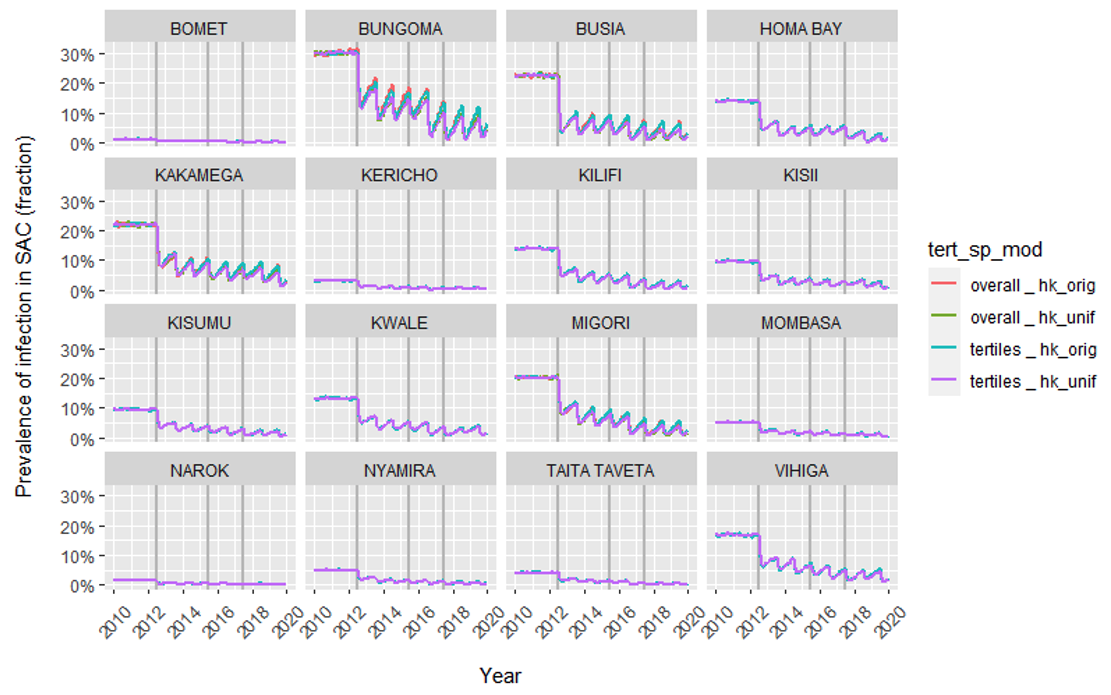

Supplement: ciae022_Supplementary_Data [file ciae022_supplementary_data.zip › SF14.png]

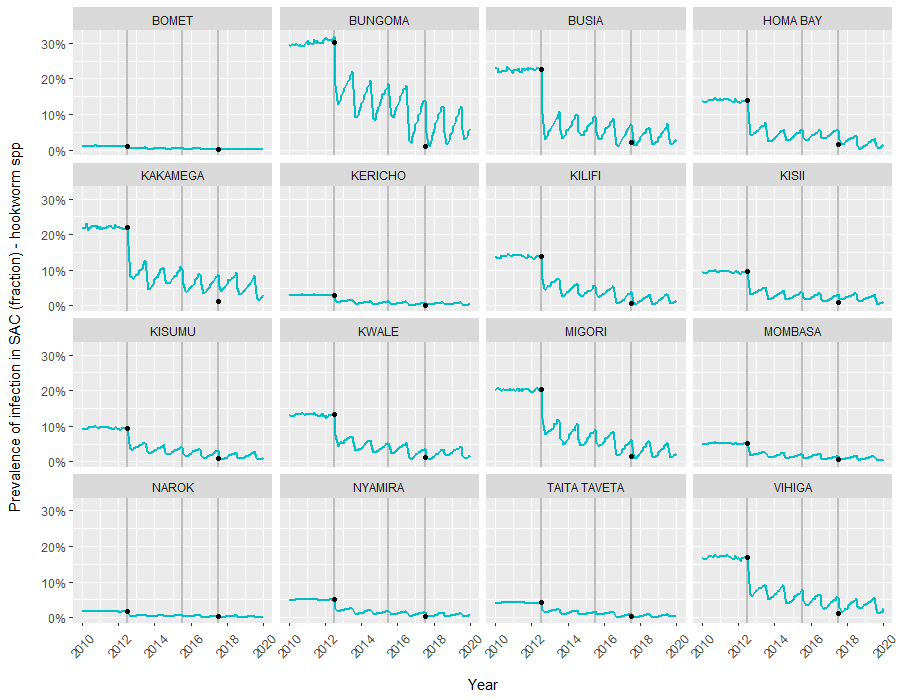

Supplement: ciae022_Supplementary_Data [file ciae022_supplementary_data.zip › SF15.png]

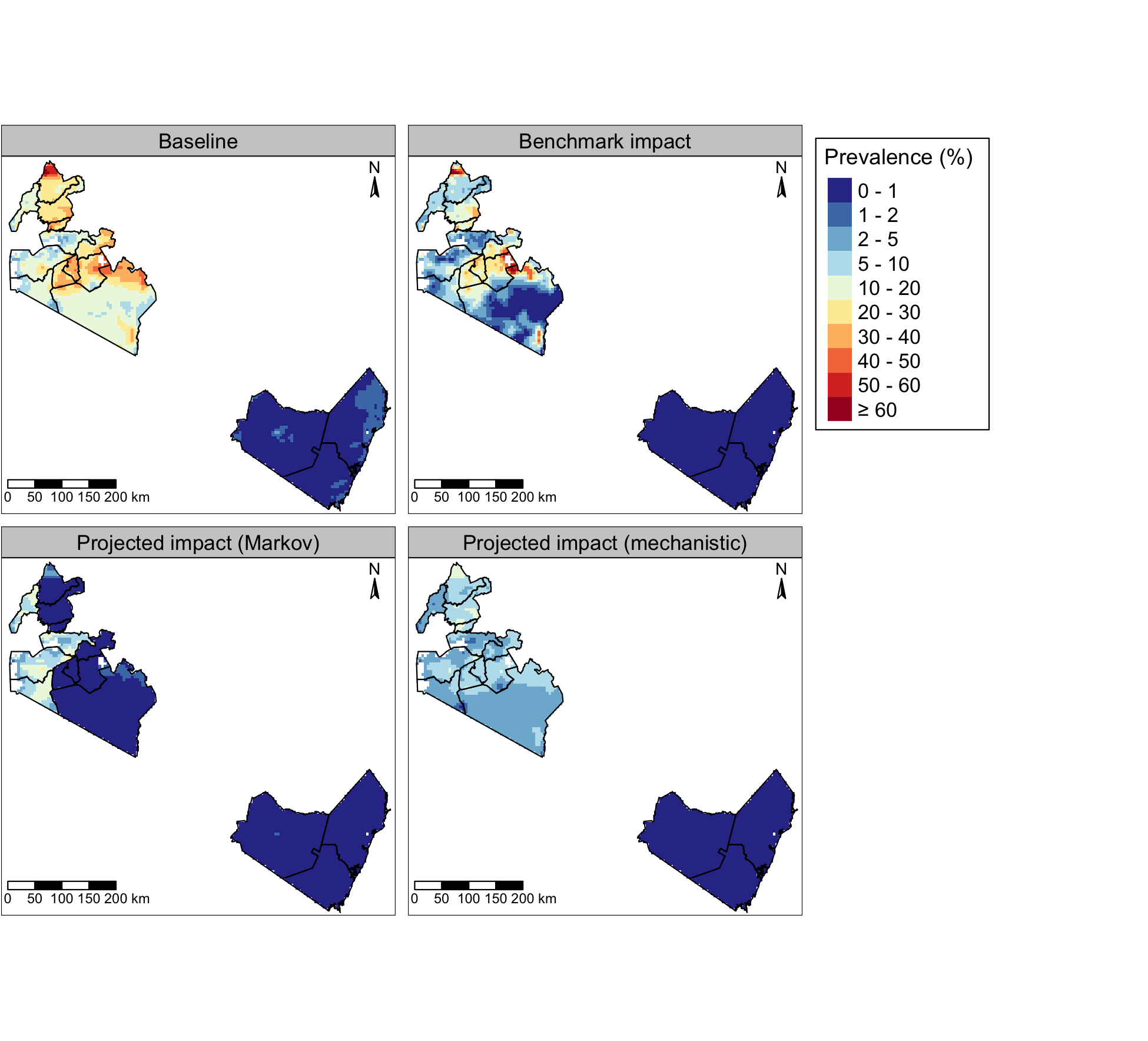

Supplement: ciae022_Supplementary_Data [file ciae022_supplementary_data.zip › SF16.png]

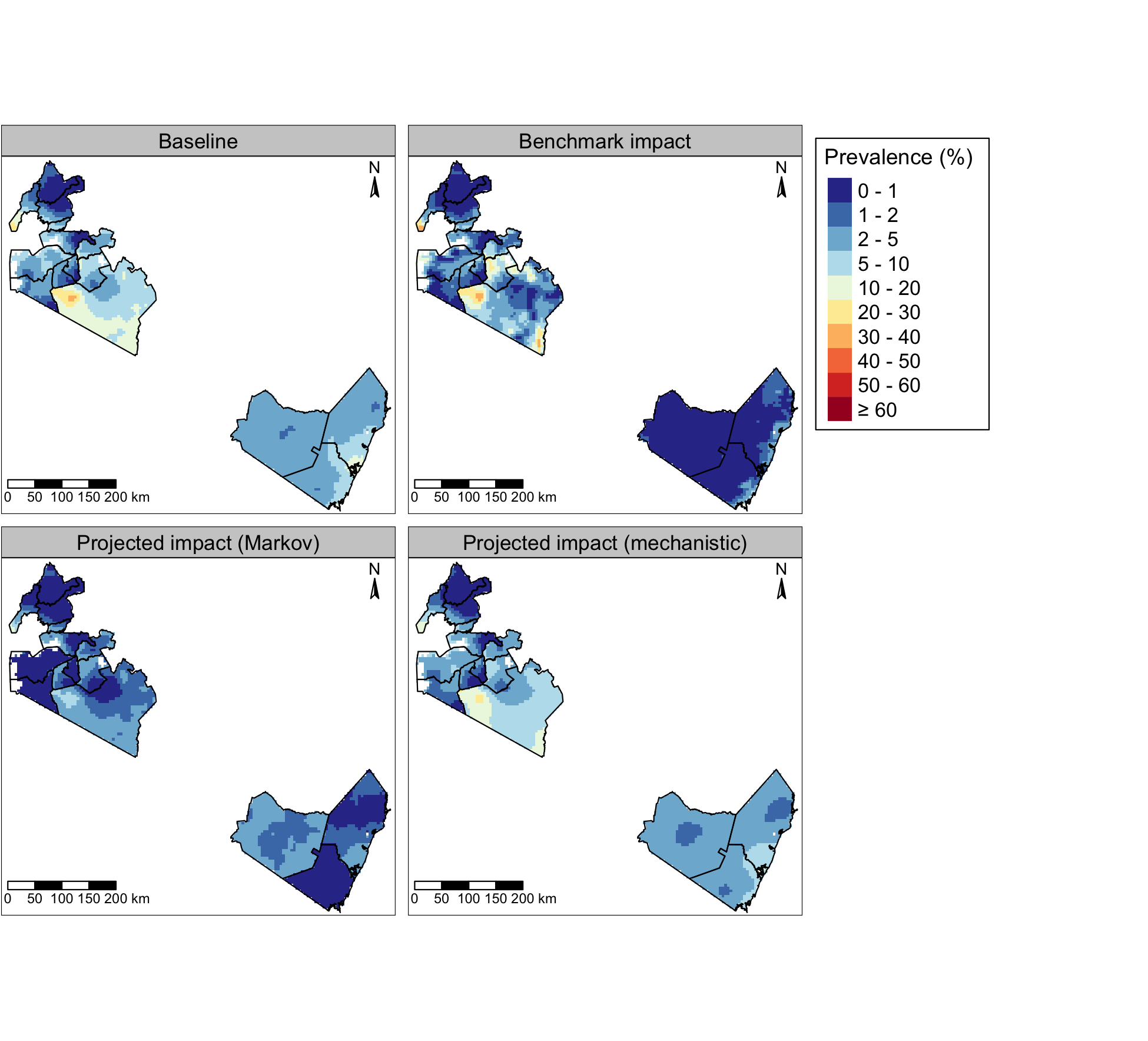

Supplement: ciae022_Supplementary_Data [file ciae022_supplementary_data.zip › SF17.png]

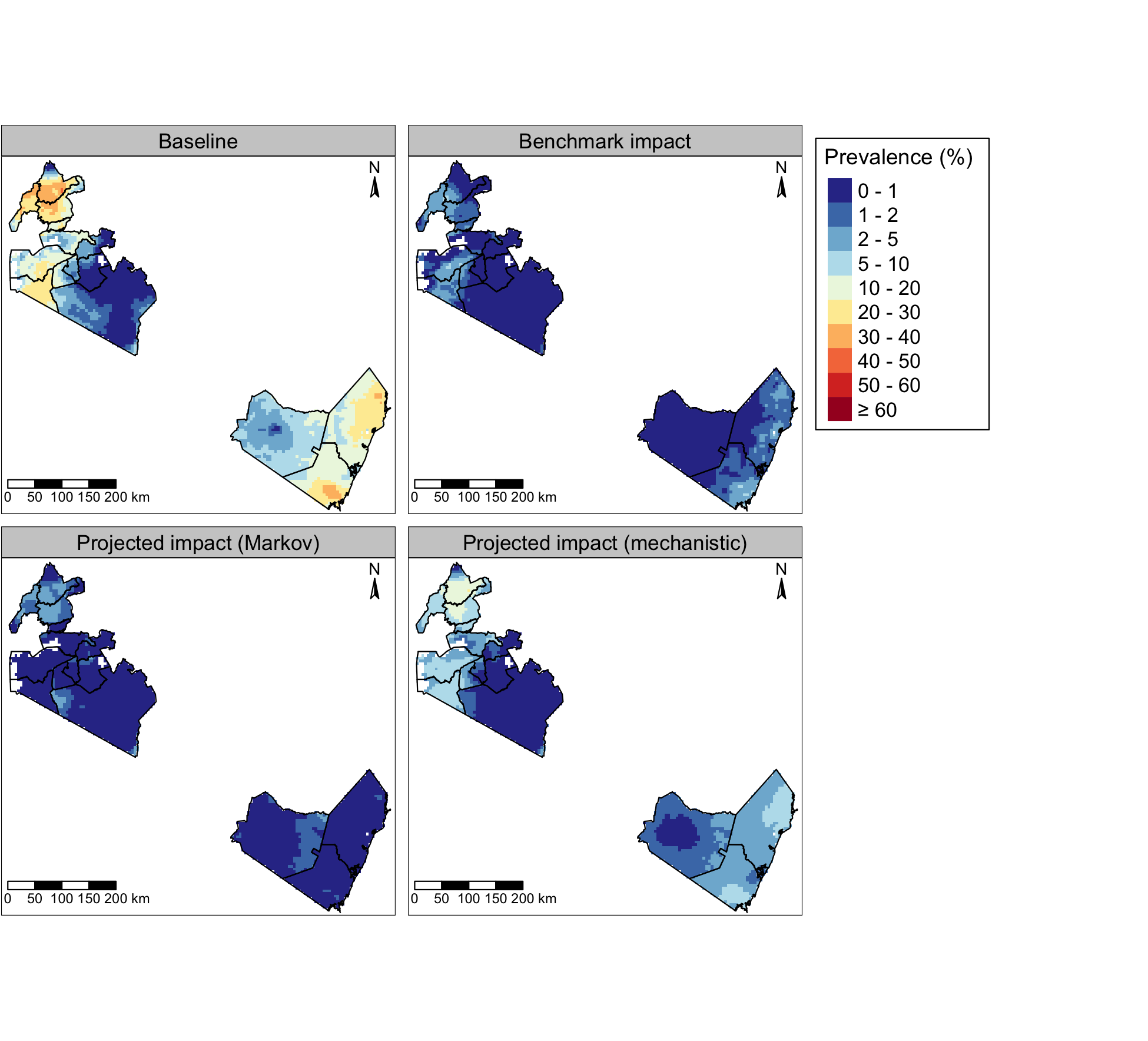

Supplement: ciae022_Supplementary_Data [file ciae022_supplementary_data.zip › SF18.png]

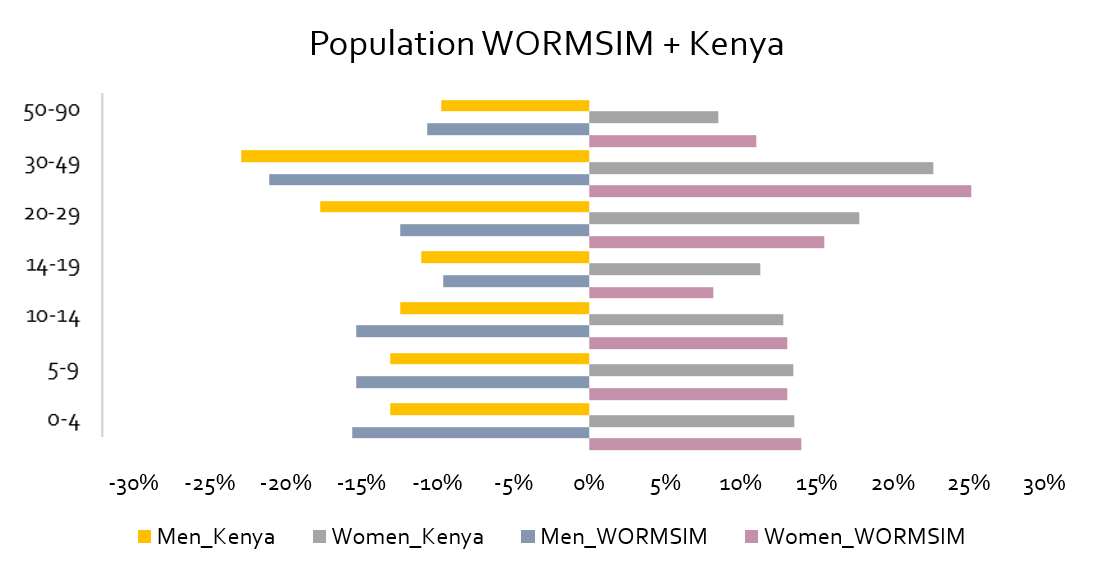

Supplement: ciae022_Supplementary_Data [file ciae022_supplementary_data.zip › SF2.1.PNG]

Log-odds of prevalence

EVI

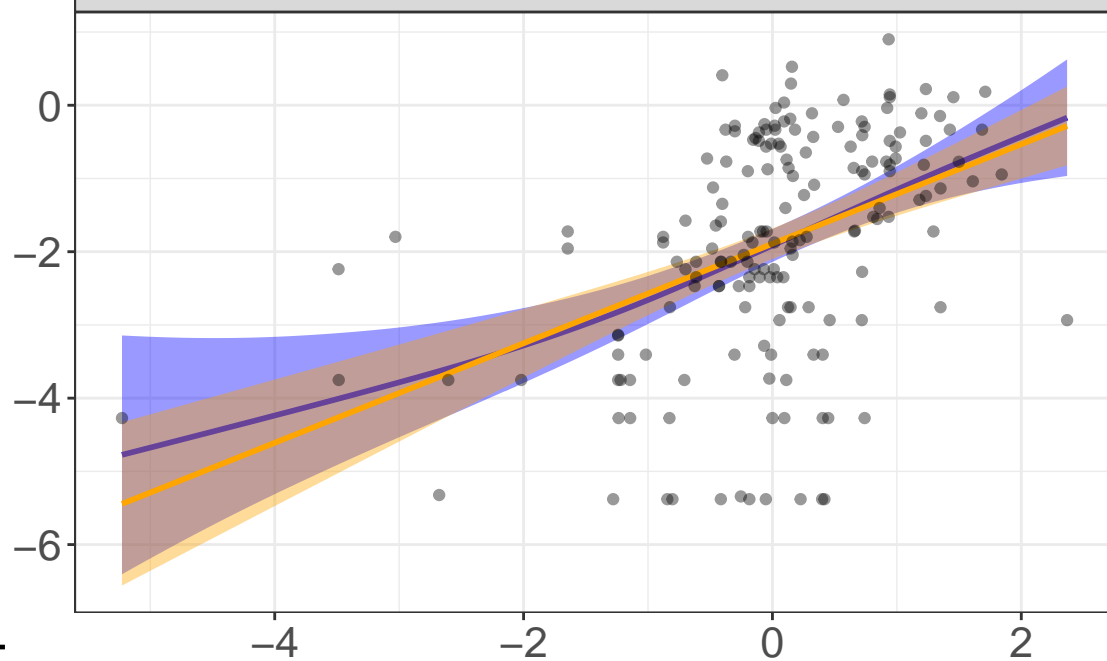

Daytime LST

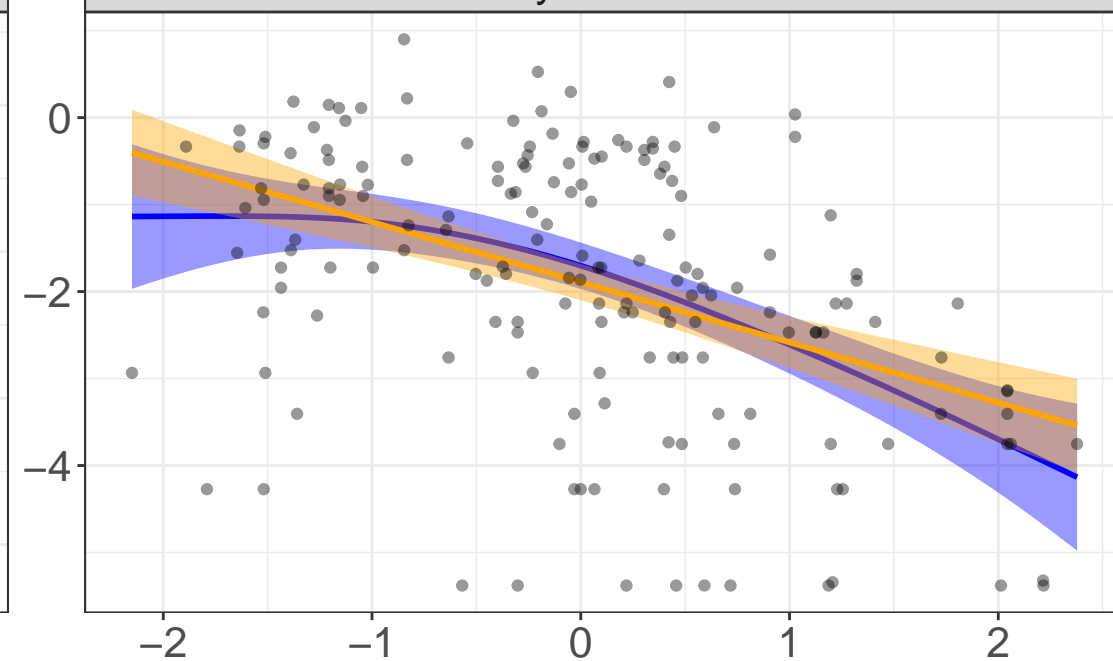

Nighttime LST

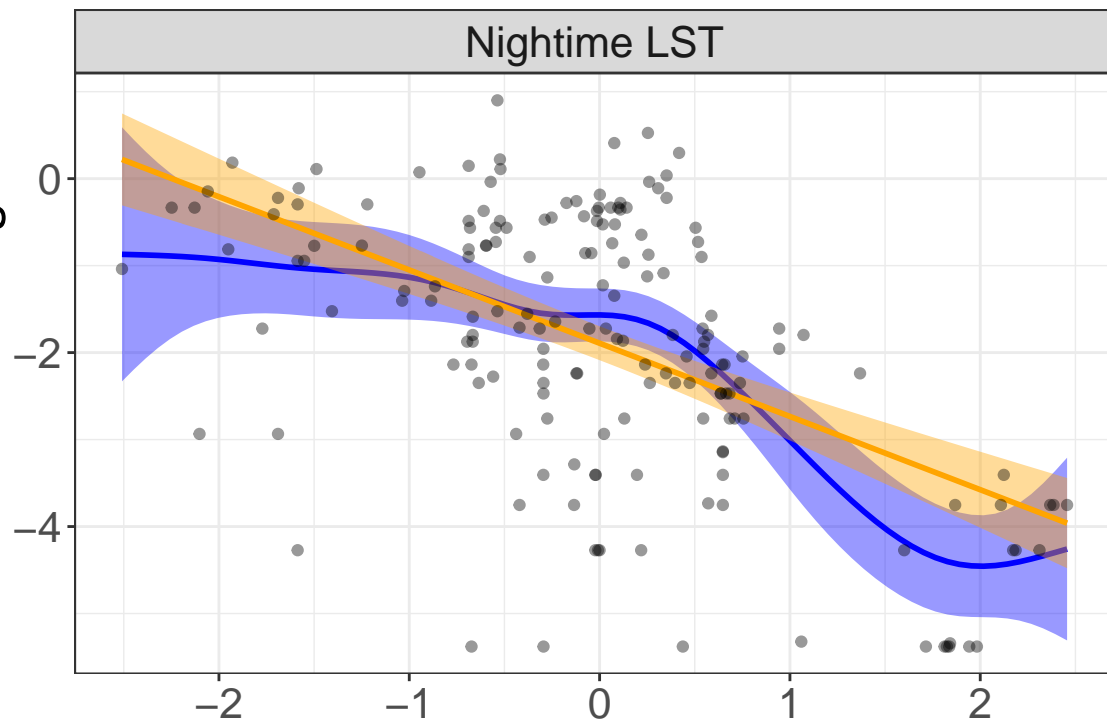

Soil acidity

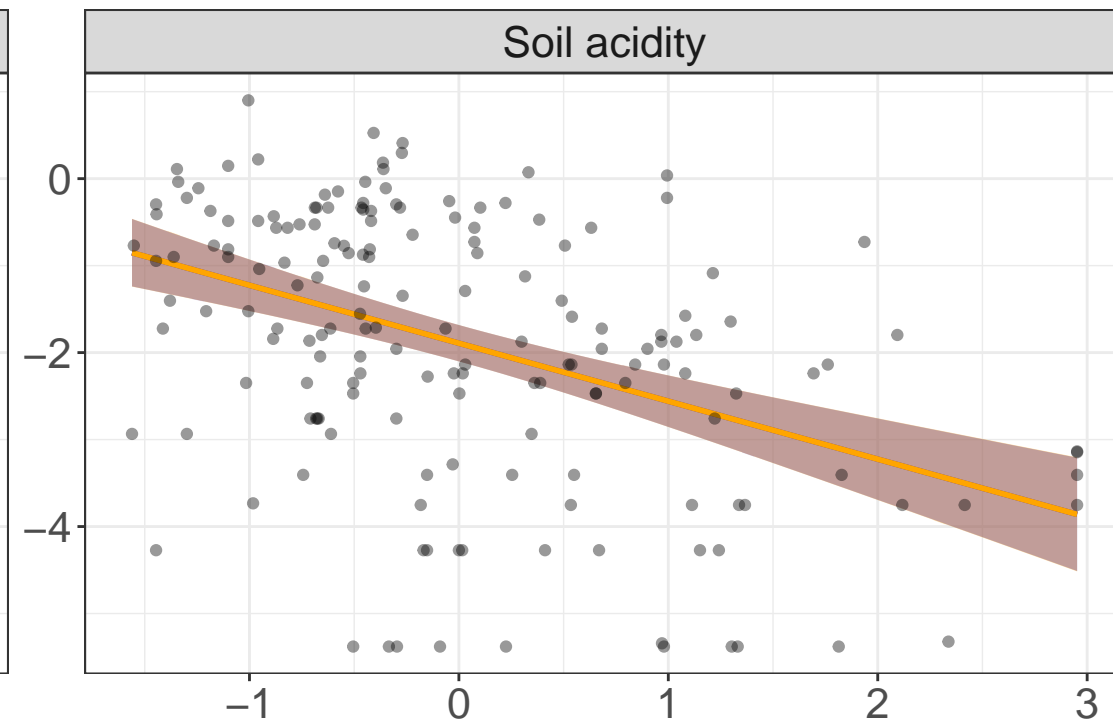

Covariate value

Supplement: ciae022_Supplementary_Data [file ciae022_supplementary_data.zip › SF3.pdf]

Log-odds of prevalence

EVI

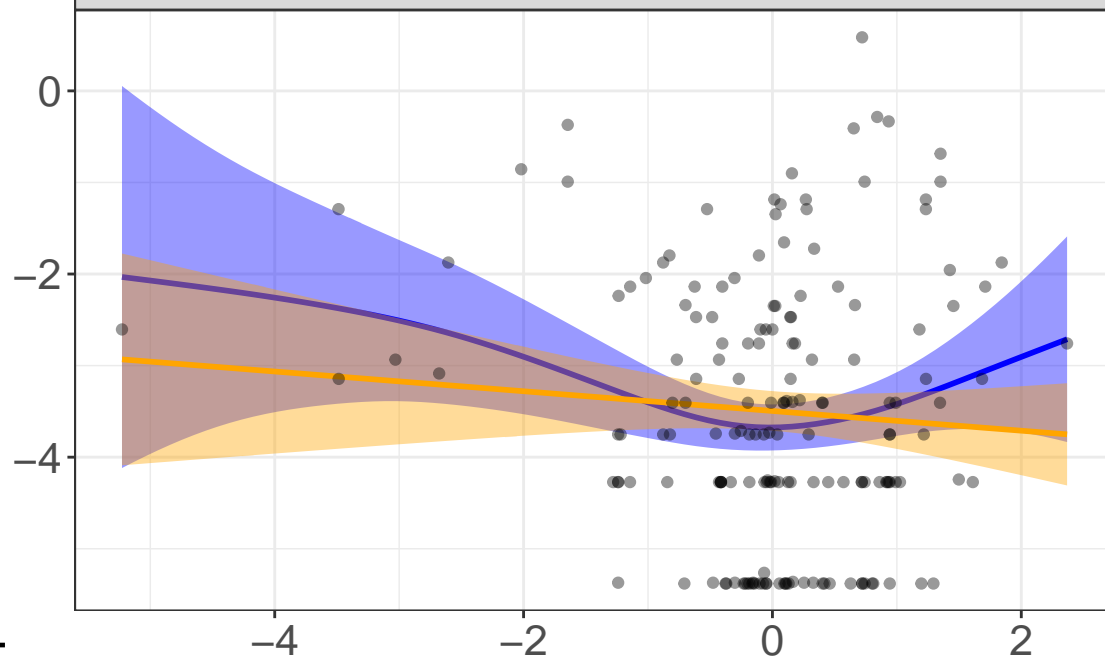

Daytime LST

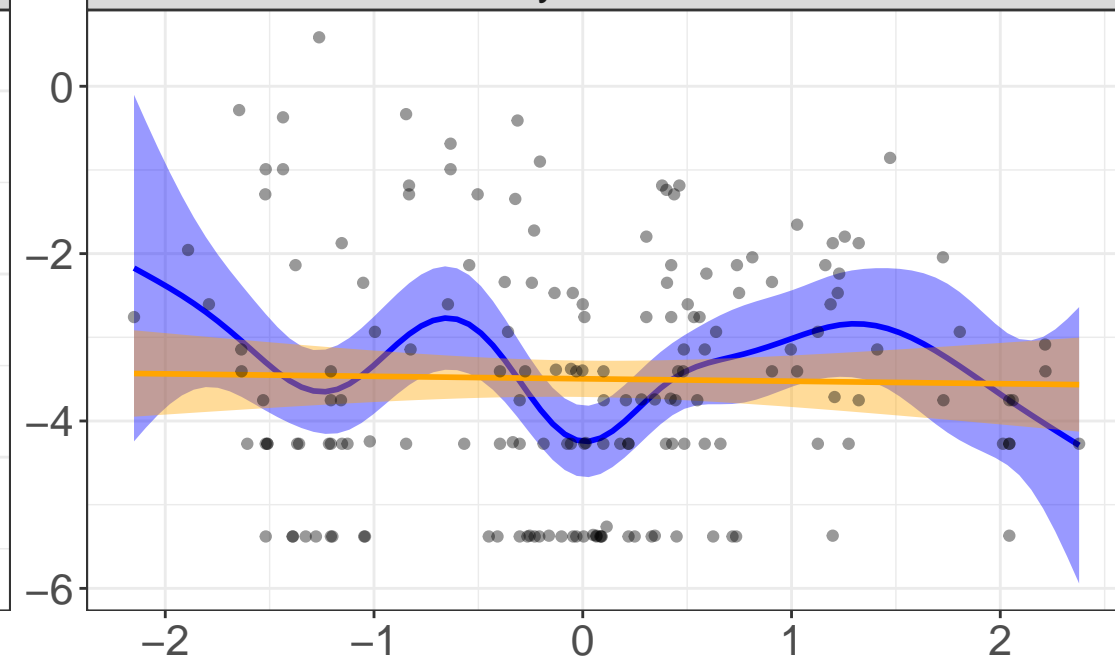

Nighttime LST

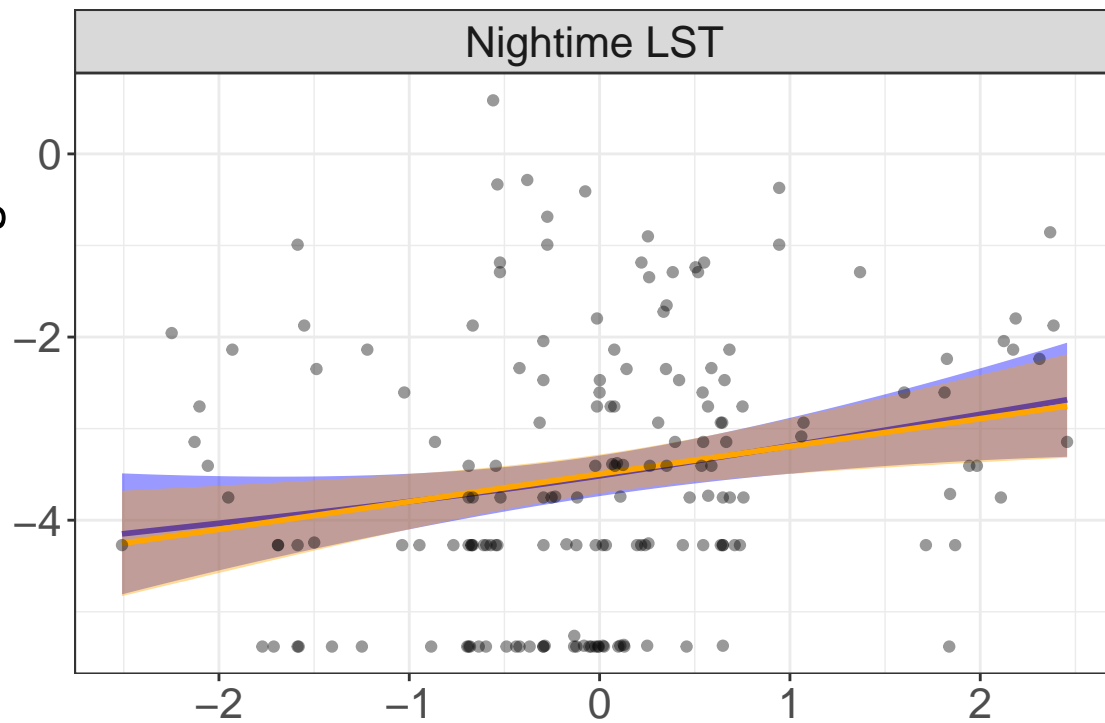

Soil acidity

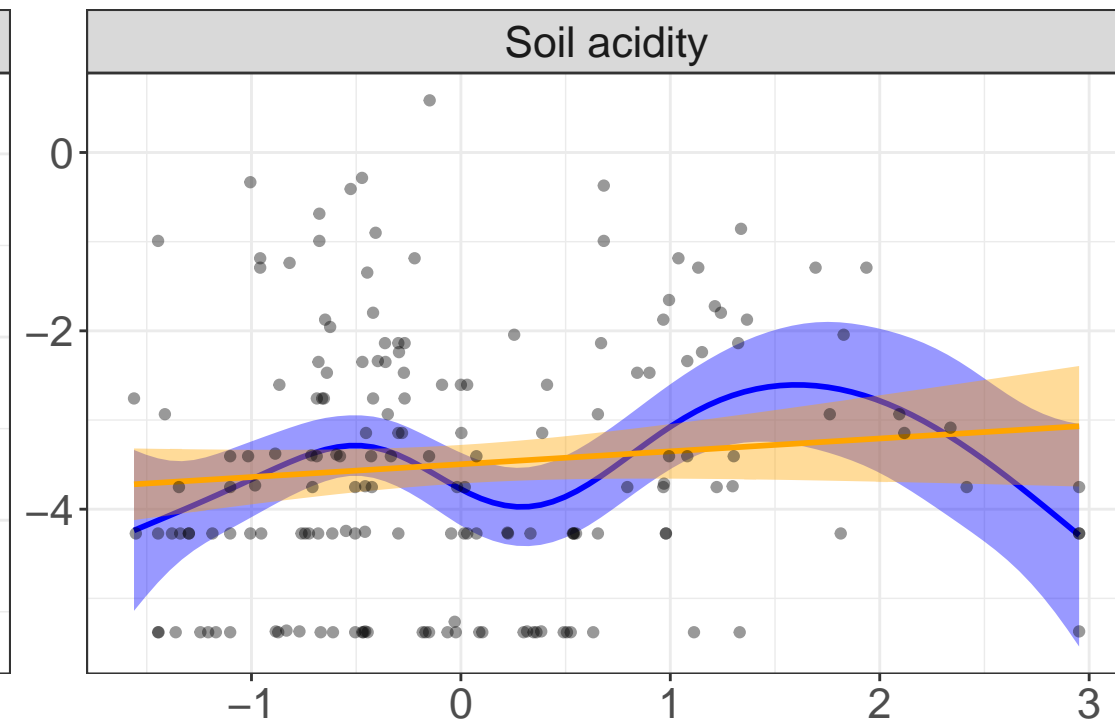

Covariate value

Supplement: ciae022_Supplementary_Data [file ciae022_supplementary_data.zip › SF4.pdf]

Log-odds of prevalence

EVI

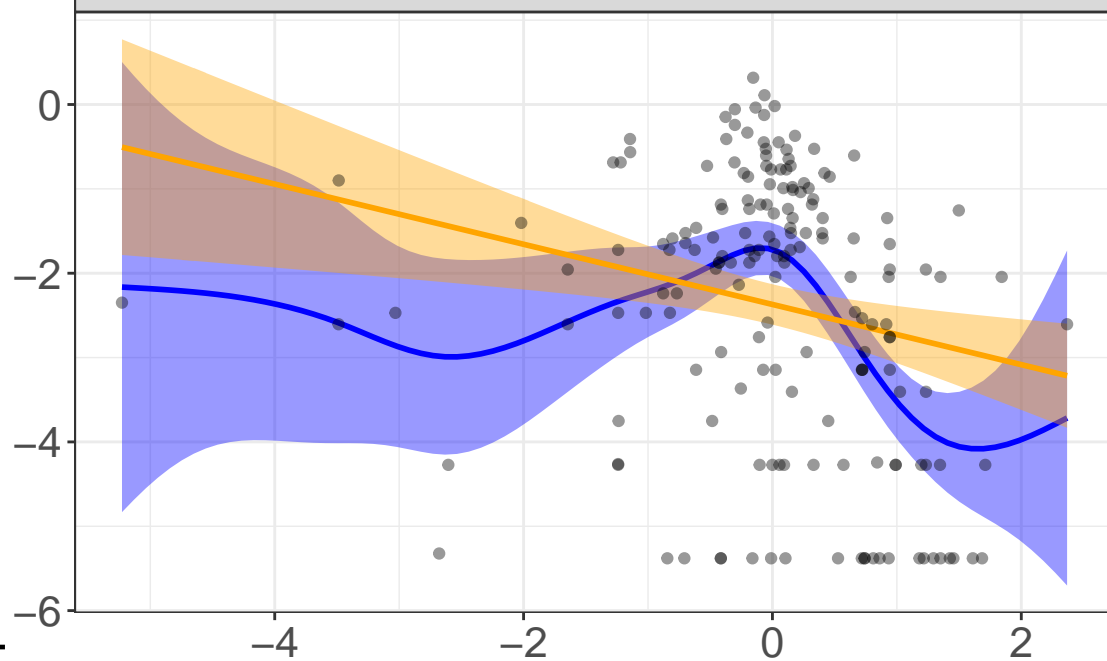

Daytime LST

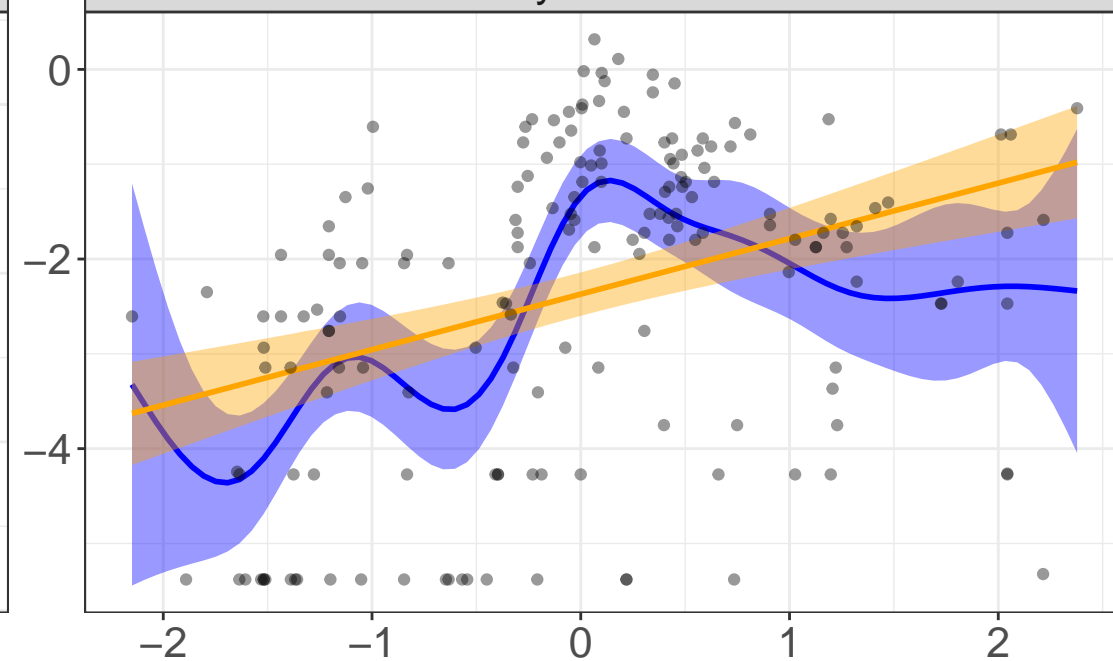

Nighttime LST

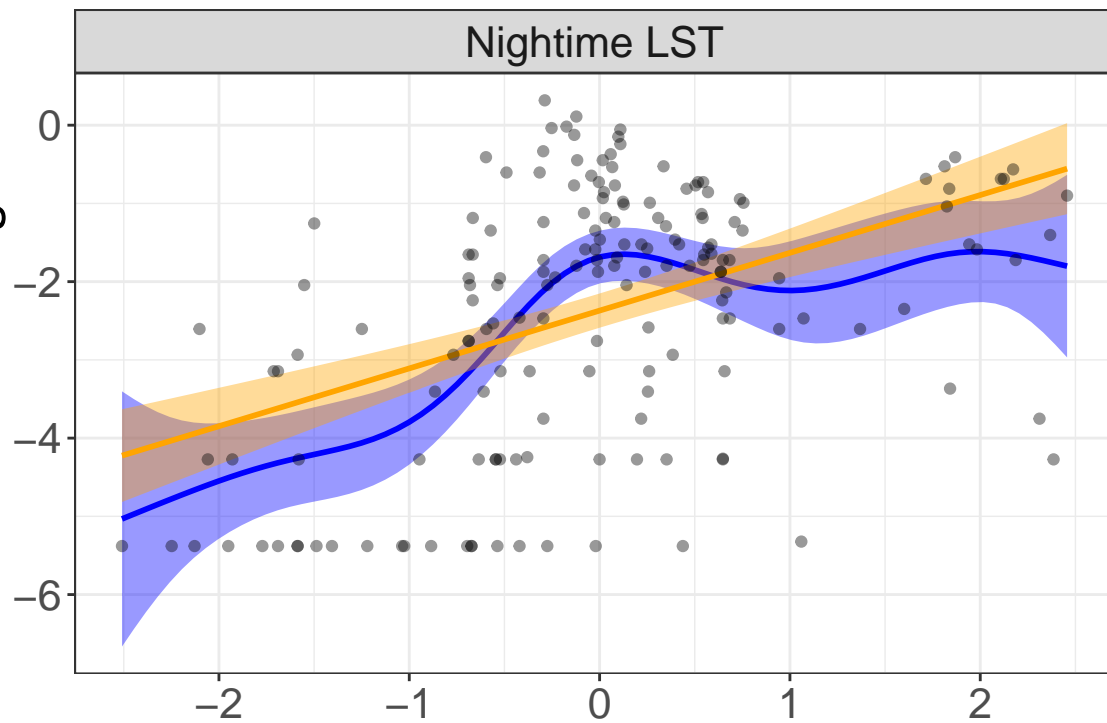

Soil acidity

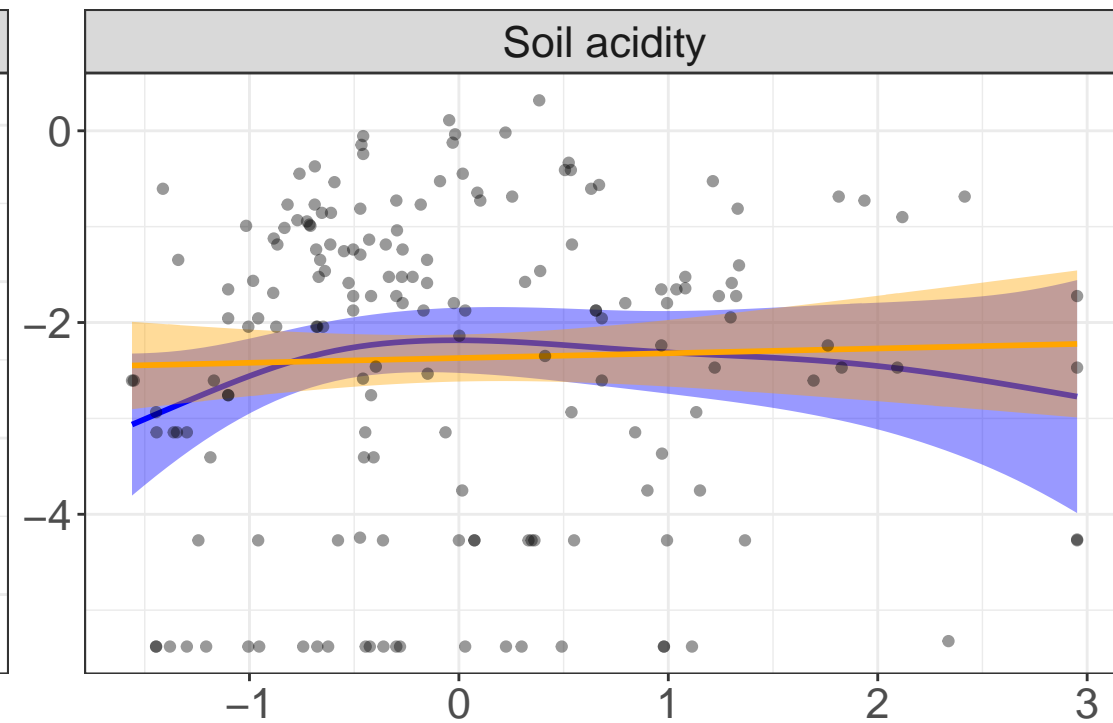

Covariate value

Supplement: ciae022_Supplementary_Data [file ciae022_supplementary_data.zip › SF5.pdf]

Log-odds of prevalence

EVI

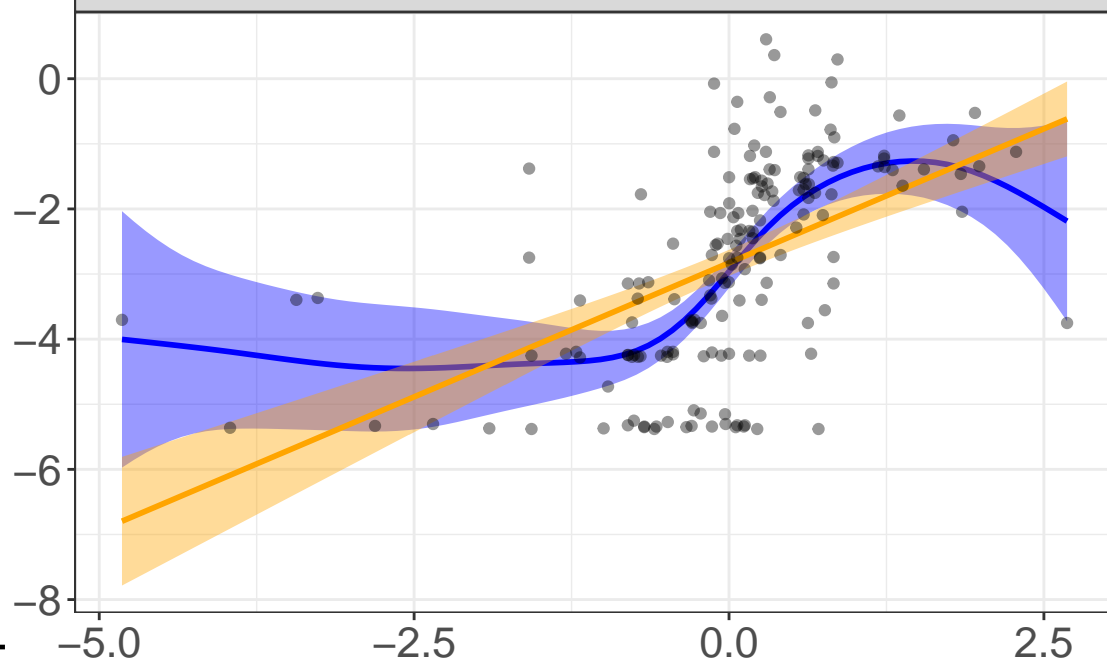

Daytime LST

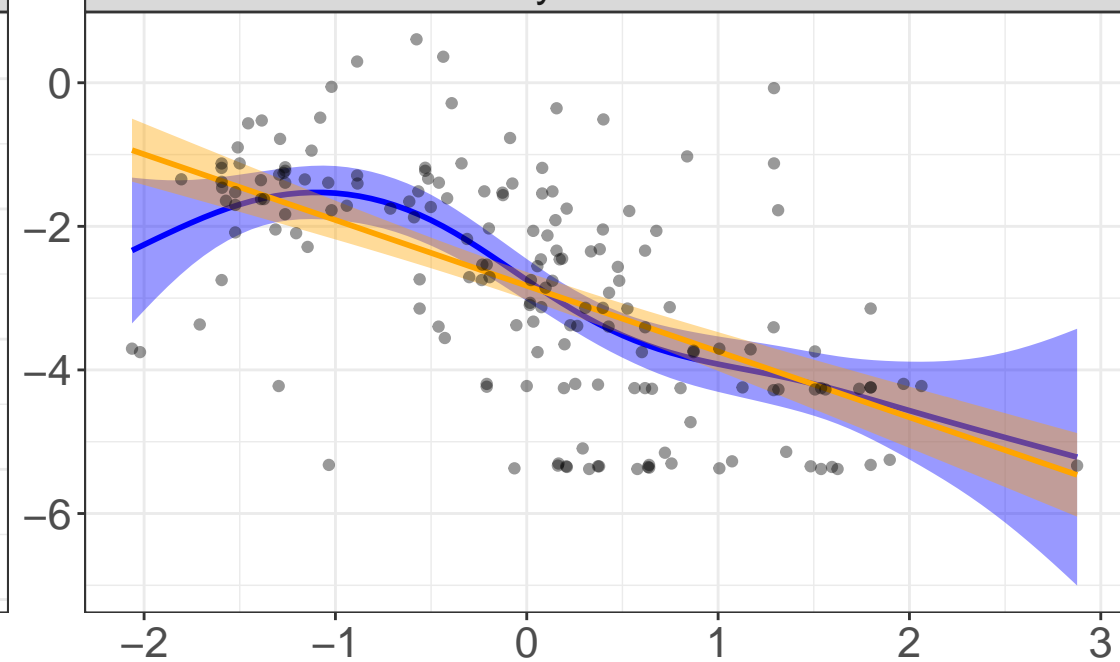

Nighttime LST

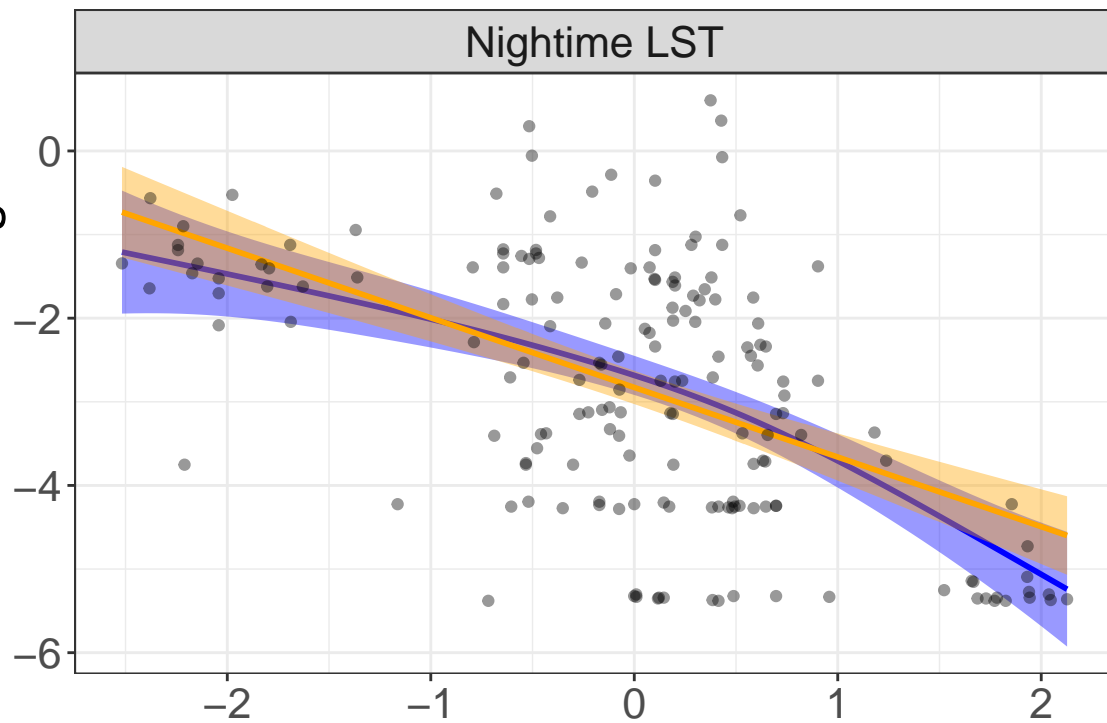

Soil acidity

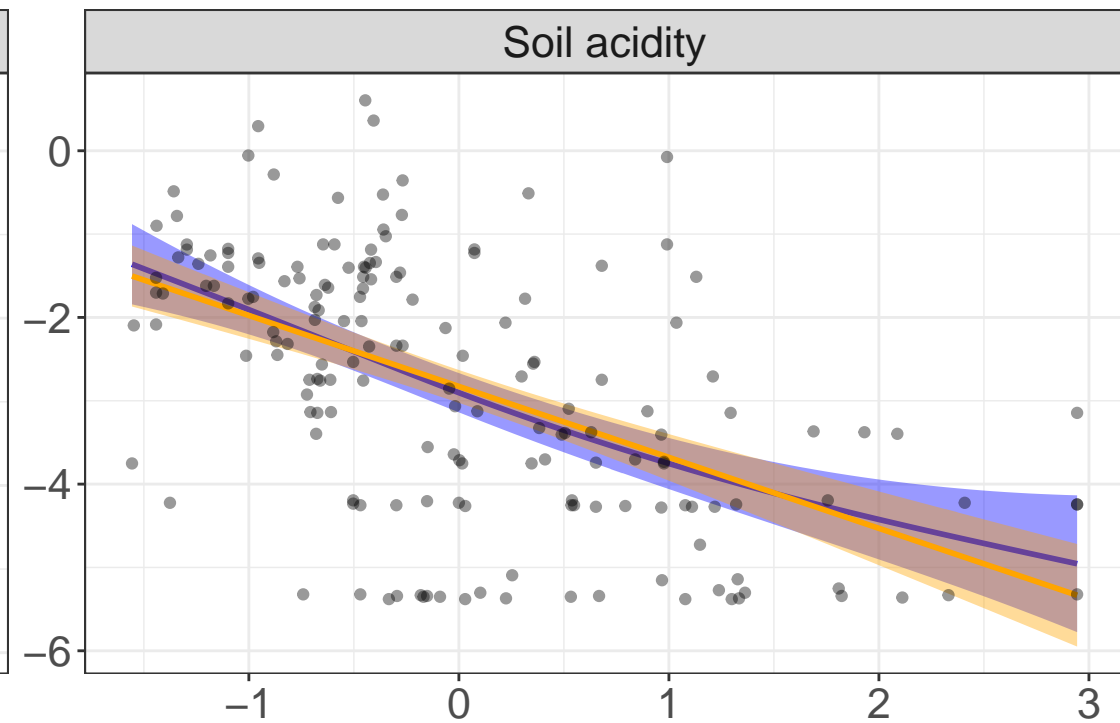

Covariate value

Supplement: ciae022_Supplementary_Data [file ciae022_supplementary_data.zip › SF6.pdf]

Log-odds of prevalence

EVI

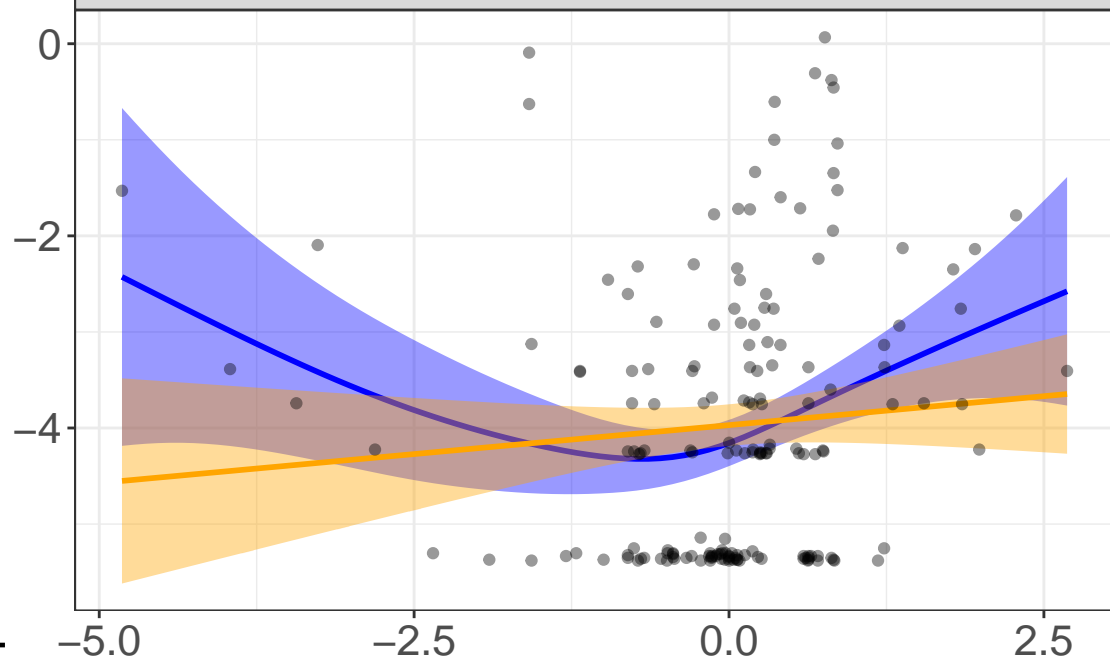

Daytime LST

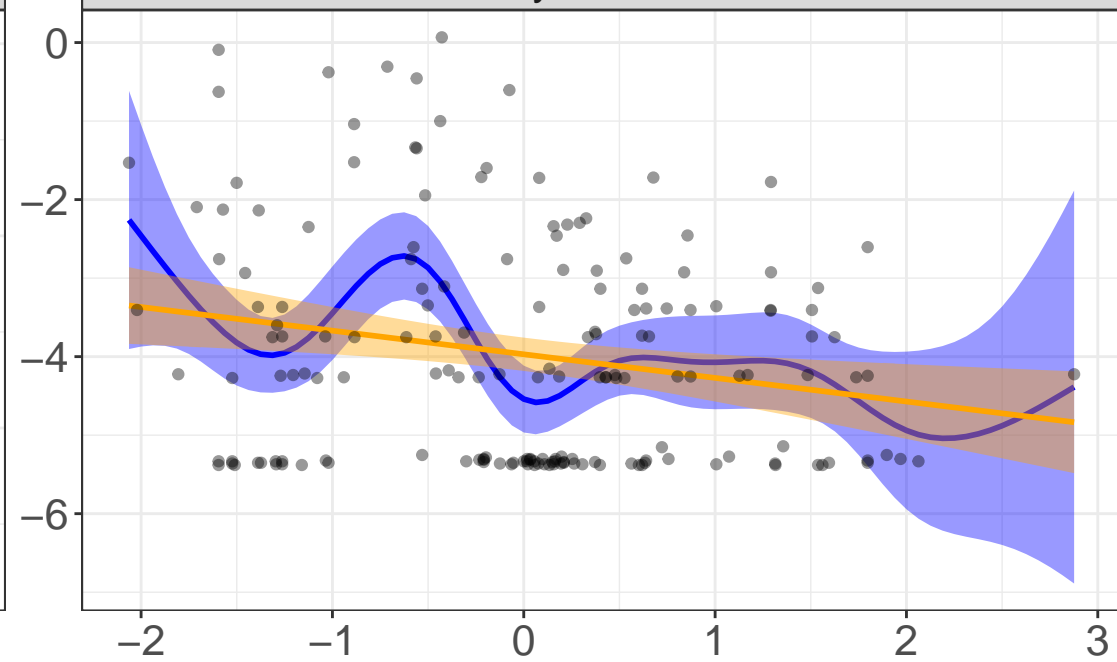

Nighttime LST

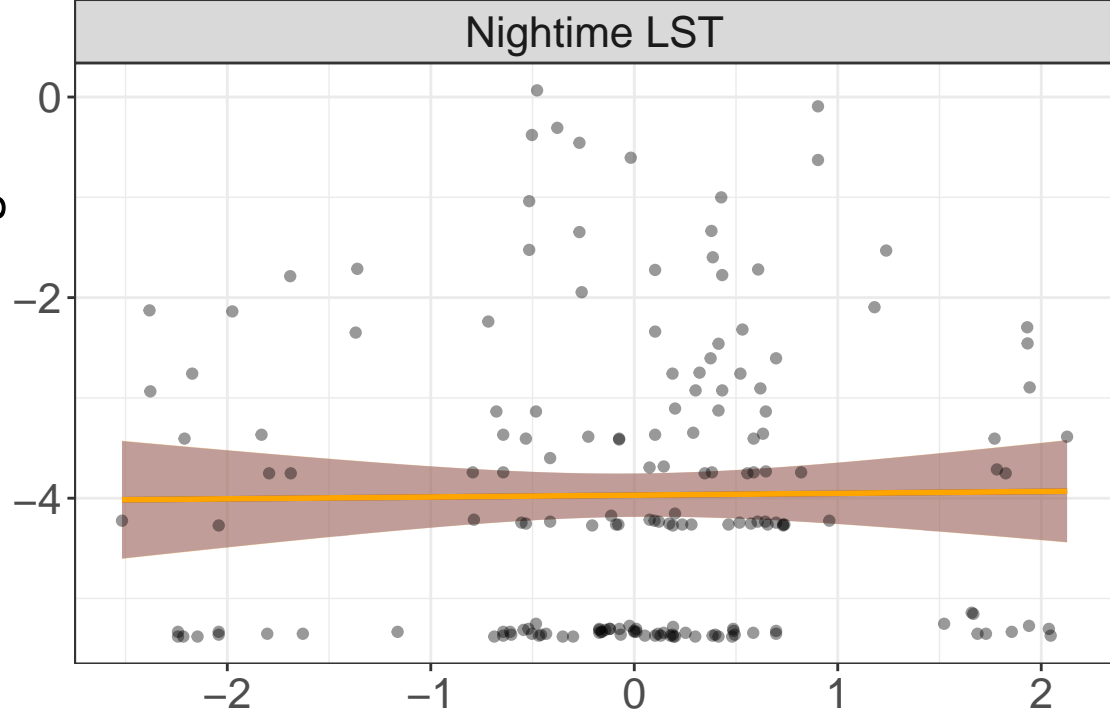

Soil acidity

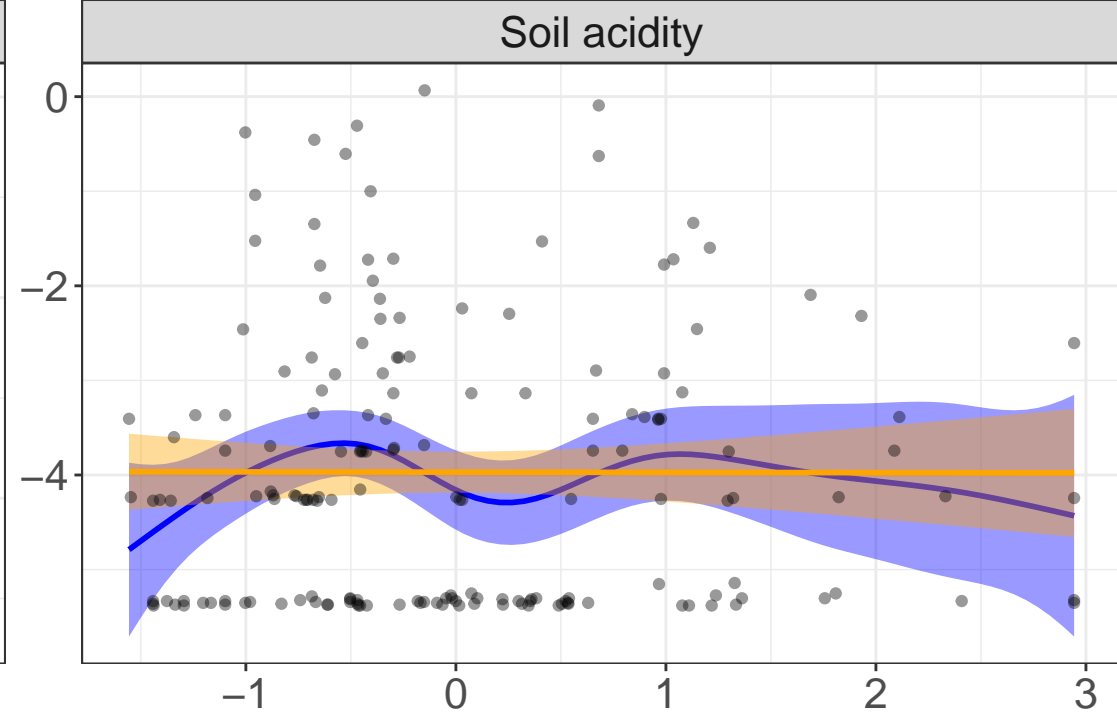

Covariate value

Supplement: ciae022_Supplementary_Data [file ciae022_supplementary_data.zip › SF7.pdf]

Log-odds of prevalence

EVI

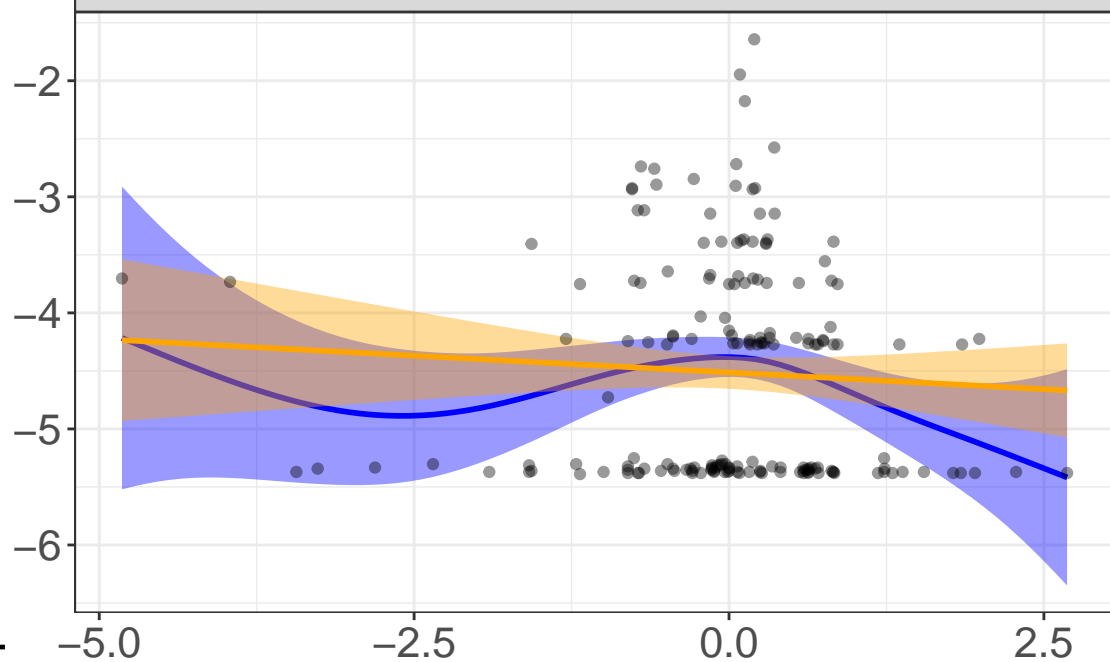

Daytime LST

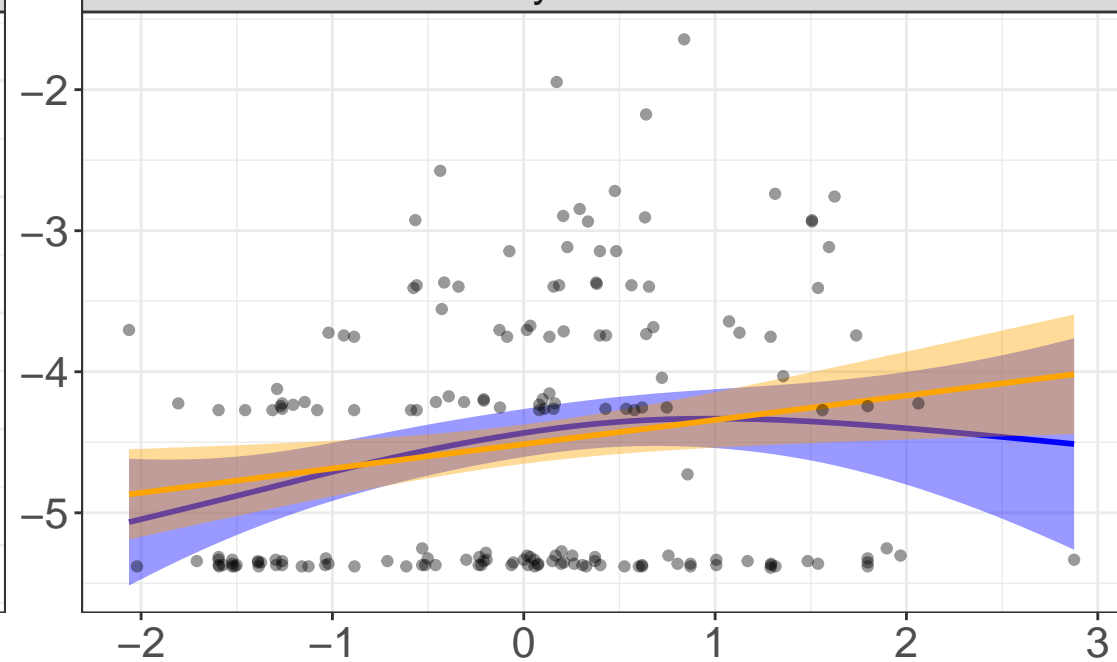

Nighttime LST

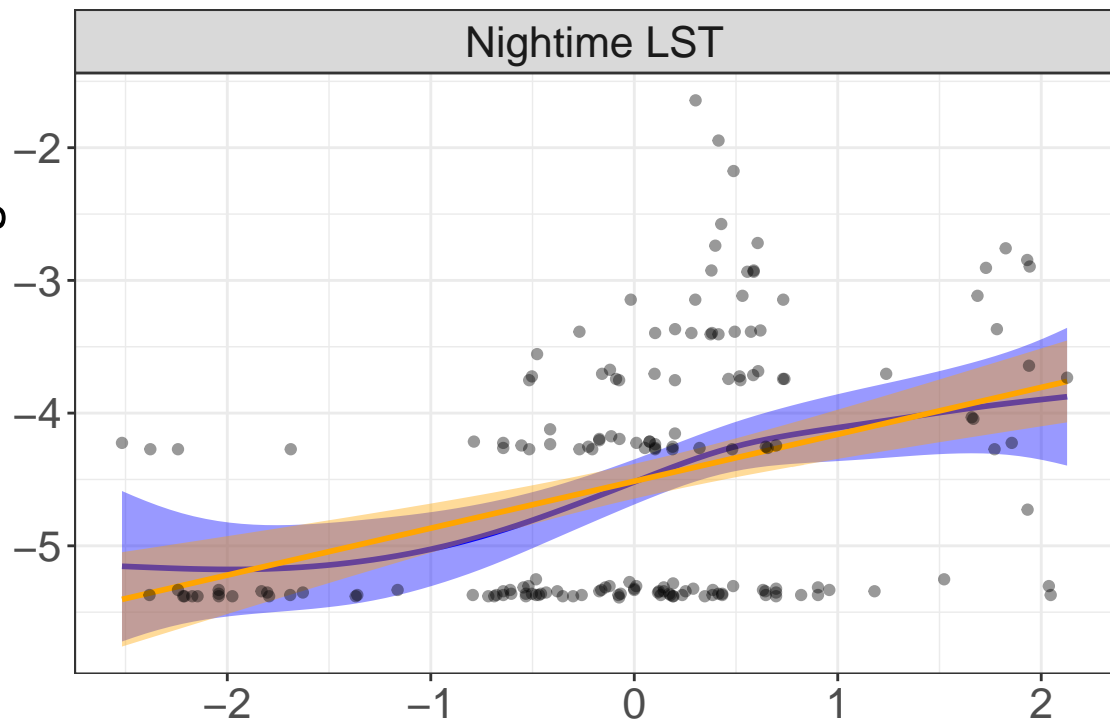

Soil acidity

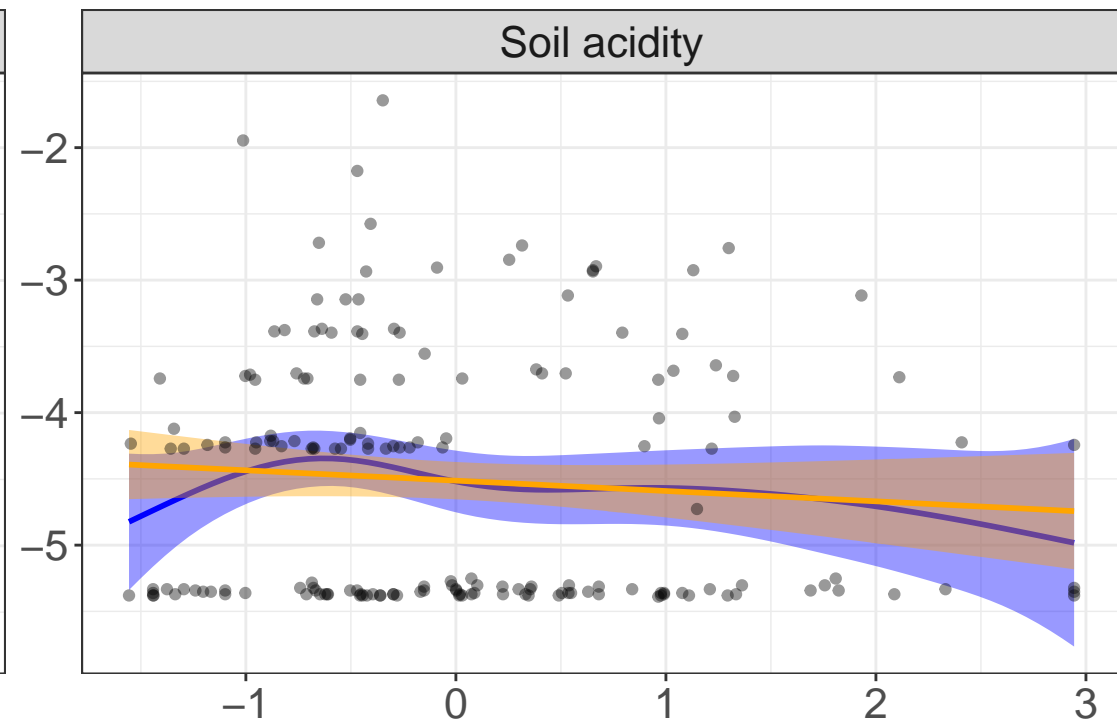

Covariate value

Supplement: ciae022_Supplementary_Data [file ciae022_supplementary_data.zip › SF8.pdf]

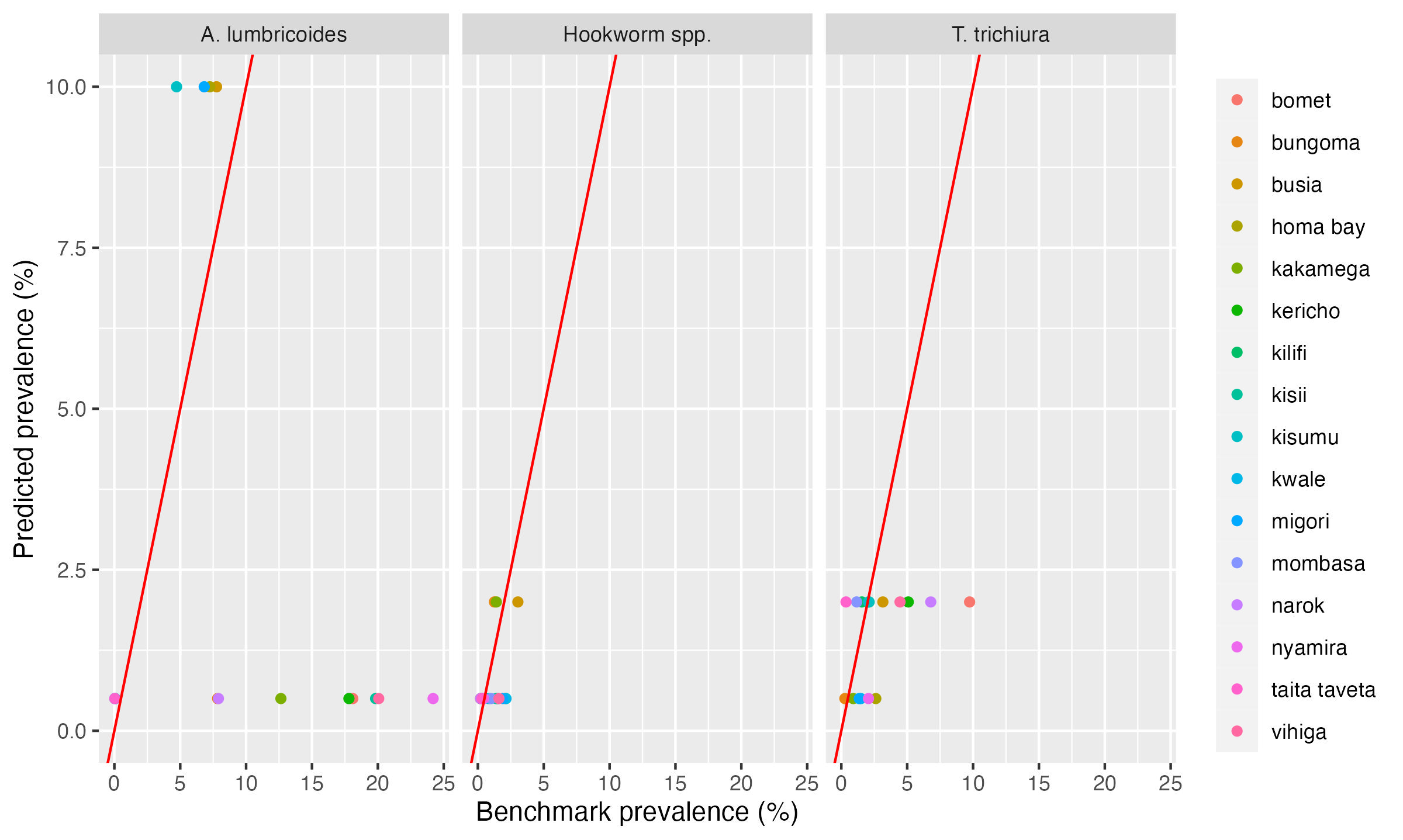

Supplement: ciae022_Supplementary_Data [file ciae022_supplementary_data.zip › SF9.jpeg]
